# An essential endoplasmic reticulum-resident N-acetyltransferase ortholog in *Plasmodium falciparum*

**DOI:** 10.1242/jcs.260551

**Published:** 2023-03-06

**Authors:** Alexander J. Polino, Muhammad M. Hasan, Katherine Floyd, Yolotzin Avila-Cruz, Yujuan Yang, Daniel E. Goldberg

**Affiliations:** Division of Infectious Diseases, Department of Medicine, and Department of Molecular Microbiology, Washington University School of Medicine, St Louis, MO 63110, USA

**Keywords:** N-terminal acetylation, Parasitology, Secretion

## Abstract

N-terminal acetylation is a common eukaryotic protein modification that involves the addition of an acetyl group to the N-terminus of a polypeptide. This modification is largely performed by cytosolic N-terminal acetyltransferases (NATs). Most associate with the ribosome, acetylating nascent polypeptides co-translationally. In the malaria parasite *Plasmodium falciparum*, exported effectors are thought to be translated into the endoplasmic reticulum (ER), processed by the aspartic protease plasmepsin V and then N-acetylated, despite having no clear access to cytosolic NATs. Here, we used inducible gene deletion and post-transcriptional knockdown to investigate the primary ER-resident NAT candidate, Pf3D7_1437000. We found that it localizes to the ER and is required for parasite growth. However, depletion of Pf3D7_1437000 had no effect on protein export or acetylation of the exported proteins HRP2 and HRP3. Despite this, Pf3D7_1437000 depletion impedes parasite development within the host red blood cell and prevents parasites from completing genome replication. Thus, this work provides further proof of N-terminal acetylation of secretory system proteins, a process unique to apicomplexan parasites, but strongly discounts a promising candidate for this post-translational modification.

## Teaser

**Summary:** An N-acetyltransferase ortholog resides in the *Plasmodium falciparum* endoplasmic reticulum and is essential for parasite survival in red blood cells, but uninvolved in protein export.

## INTRODUCTION

N-terminal acetylation is among the most common modifications to eukaryotic proteins (Deng and Marmorstein, 2021). Typically during or soon-after translation, an acetyl group is transferred from acetyl-CoA to the N-terminus of a polypeptide. This alteration blunts the N-terminal charge, changing its chemical properties and altering the way the polypeptide interacts with various biological systems. In some cases, N-terminal acetylation changes a protein's half-life (Hwang et al., 2010; Park et al., 2015). In others, proper interaction with binding partners relies on acetylation (Scott et al., 2011). Acetylation is performed by N-terminal acetyltransferases (NATs), nearly all of which are cytosolic enzymes that typically associate with the ribosome. In eukaryotes, eight currently known NAT complexes combine to acetylate most cytosolic N-termini, as well as some proteins in the chloroplast lumen.

The malaria parasite *Plasmodium falciparum* marks effectors for export into the host cell with a pentameric amino acid sequence called the *Plasmodium* export element (PEXEL) (Hiller et al., 2004; Marti et al., 2004). PEXEL-containing proteins are believed to be translated into the parasite endoplasmic reticulum (ER), where PEXEL is cleaved by the aspartic protease plasmepsin V (PM V; Pf3D7_1323500) (Boddey et al., 2010; Russo et al., 2010) after a conserved leucine. Previous work with exported reporters revealed that, following PEXEL cleavage in the ER, the new N-terminus is somehow acetylated (Boddey et al., 2009; Chang et al., 2008; Osborne et al., 2010). This acetylation occurred even if exported proteins were sequestered in the ER by brefeldin A treatment (blocking anterograde traffic from the ER) or addition of an ER retention signal (Chang et al., 2008; Osborne et al., 2010). This suggests that an as-yet unidentified NAT exists in the *P. falciparum* ER. Subsequently Tarr et al. mutagenized the PEXEL motif of the exported protein REX3 and found that point mutants that were not acetylated also were not exported (Tarr et al., 2013), demonstrating coincidence between these two processes. The identity of the PEXEL NAT in *P. falciparum*, and its role in export, if any, remains to be determined. In the related parasite *Toxoplasma gondii,* extensive N-terminal acetylation of proteins in the secretion pathway at the ER has recently been reported along with the presence of an ER-resident NAT, TgNAT8 (Nyonda et al., 2022).

Here, we searched the genome for putative NATs in *P. falciparum* that might access the secretory system. We identified Pf3D7_1437000, the homolog of TgNAT8, as the most likely candidate for follow-up investigation. Depletion of Pf3D7_1437000 arrested parasite growth in culture, but had no detectable effect on protein export or on exported protein N-terminal acetylation. The phenotype manifested by Pf3D7_1437000 depletion is distinct from that seen after disruption of an essential component of the export pathway, suggesting that the essential role of Pf3D7_1437000 is not in facilitating protein export. Instead, Pf3D7_1437000 depletion resulted in parasites with reduced size that failed to complete genome replication.

## RESULTS

**Fig. 1.**
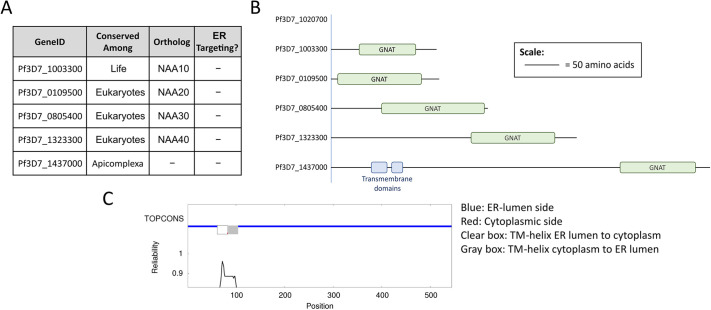
**Putative protein N-acetyltransferases encoded by *P. falciparum*.** (A) Table of putative N-acetyltransferases (NATs) in the *P. falciparum* genome. Genes were included based on assignment to the GNAT enzyme superfamily in PlasmoDB, and subsequently excluded if they had a different predicted function (see text). Orthology was based on grouping in OrthoMCL and supported by reciprocal BLAST searches. Endoplasmic reticulum (ER)-targeting sequences were sought with SignalP 4.1, and transmembrane domains (TM) were annotated based on TransMembrane prediction using Hidden Markov Models (TMHMM) predictions. (B) Diagram of the identifiable domains in the NAT candidates in A. Diagram is to scale as shown. (C) Topcons consensus-predicted membrane topology for Pf3D7_1437000.

To search for an ER-resident NAT, we used PlasmoDB (Amos et al., 2022) to identify *P. falciparum* genes for which the sequence was annotated to contain a motif assigning them to the GNAT enzyme superfamily. This search yielded eight genes. Two – Pf3D7_ 0823300 and Pf3D7_0629000 – have previously been studied in *P. falciparum* and are proposed to act as a GCN5 histone acetyltransferase and a glucosamine-phosphate N-acetyltransferase, respectively (Cova et al., 2018; Fan et al., 2004). Five of the remaining six have orthologs in metazoans. Pf3D7_1020700 appears similar to human NAT10, an RNA cytidine acetyltransferase (Ito et al., 2014). Pf3D7_1003300, Pf3D7_0109500, Pf3D7_0805400 and Pf3D7_1323300 are the closest orthologs of the cytosolic human N-acetyltransferases NAA10, NAA20, NAA30 and NAA40, respectively (Fig. 1A) (Chen et al., 2006). The remaining candidate NAT, Pf3D7_1437000, appears to have orthologs among the Apicomplexa, but no obvious representative outside the phylum. None of the candidate NATs had an apparent signal or retention sequence to drive ER localization. However, Pf3D7_1437000 had two predicted transmembrane domains (Fig. 1B), making it the likeliest candidate to access the secretory system. Its homolog in *T. gondii* is likely localized in the ER membrane with the GNAT domain residing inside the ER lumen (Nyonda et al., 2022). Membrane topology prediction using the freely available program TOPCONS (Tsirigos et al., 2015) placed the GNAT domain of Pf3D7_1020700 also within the ER lumen (Fig. 1C; Fig. S1). With that in mind, we focused our efforts on characterizing Pf3D7_1437000.

**Fig. 2.**
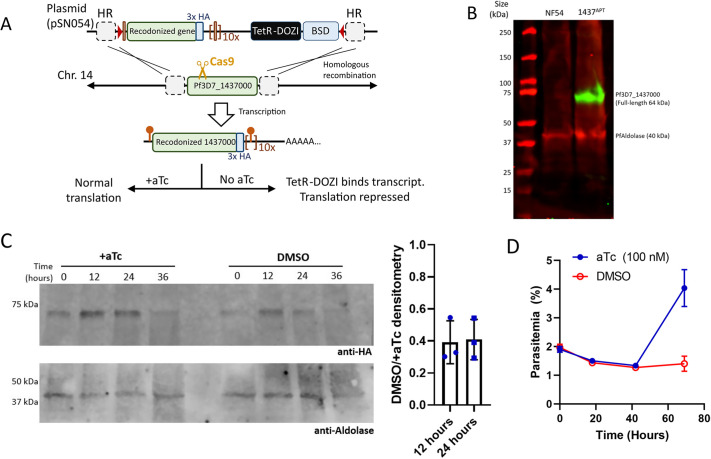
**Tetracycline repressor-aptamer system for inducible Pf3D7_1437000 depletion.** (A) Diagram of genome editing and inducible depletion of Pf3D7_1437000. The endogenous gene was disrupted by Cas9 and replaced by a recodonized version flanked with aptamers (orange circles with stems) and loxP sites (red triangles). When transcribed, the aptamers fold to bind TetR. In the absence of anhydrotetracycline (aTc), TetR binds the aptamers; its fusion protein DOZI sequesters the bound mRNA, repressing translation. BSD, blasticidin deaminase; HR, homologous region. (B) Western blot probing lysate from the parent (NF54) or tagged line (1437^APT^) with anti-HA (green) and anti-*P. falciparum* fructose-bisphosphate aldolase (PfAldolase, red). (C) Left: parasites were synchronized at the ring stage and incubated in medium containing 100 nM aTc (+aTc) or an equal volume of DMSO (DMSO). Samples were lysed at 12-h intervals and probed for Pf3D7_1437000-HA and PfAldolase. Right: densitometry was used to quantify the fraction of Pf3D7_1437000-HA in the DMSO versus aTc sample over three independent experiments. (D) Parasites were grown as above and their growth was monitored daily by flow cytometry. The experiment was performed three times, with each culture in technical triplicate. A representative experiment is shown: points represent the mean of technical triplicates, error bars represent the s.d. of those measurements. Uncut gel for panel C is shown in Fig. S3.

We targeted Pf3D7_1437000 using CRISPR/Cas9 editing and the previously described pSN054 vector to replace the gene with a recodonized version that is C-terminally hemagglutinin (HA) tagged and flanked with loxP sites for gene excision, as well as tetracycline repressor (TetR)-binding aptamers for post-transcriptional depletion (Fig. 2A) (Polino et al., 2020). Proper genome editing was confirmed by Southern blotting (Fig. S2). Western blotting for HA revealed a single band consistent with tagged full-length Pf3D7_1437000 (expected size 64 kDa) (Fig. 2B). Parasite lysates taken at 12-h intervals across the intraerythrocytic development cycle revealed that Pf3D7_1437000 was present throughout the cycle, although predominantly at the 12- and 24-h time points (Fig. 2C; Fig. S3). When Pf3D7_1437000 depletion was induced by maintaining synchronized parasites in media without anhydrotetracycline (aTc), Pf3D7_1437000 levels fell to approximately 40% of the levels in the presence of 100 nM aTc (+aTc) within 12 h (Fig. 2C). This resulted in parasite death within a single intraerythrocytic development cycle, showing Pf3D7_1437000 to be essential for growth in parasite culture (Fig. 2D; Fig. S3).

### Pf3D7_1437000 localizes to the ER

**Fig. 3.**
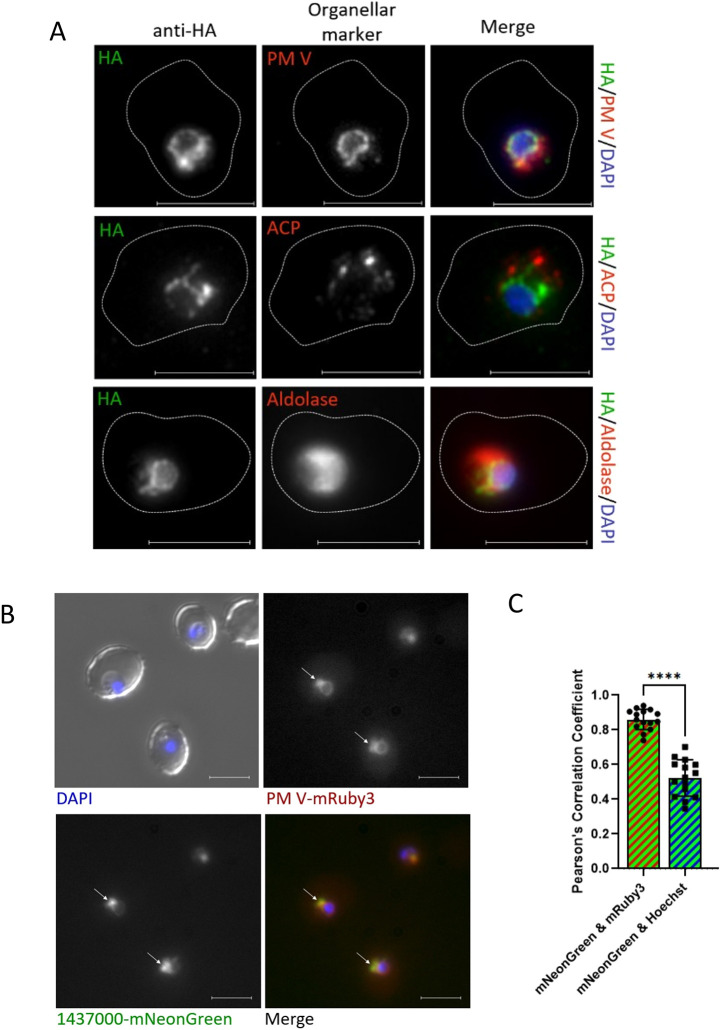
**Pf3D7_1437000 resides in the ER.** (A) Micrographs of immunofluorescence assay to localize Pf3D7_1437000-HA (green in merge) alongside plasmepsin V (PM V), ACP or aldolase (red in merge). Experiment was performed three times; representative images are shown. Scale bars: 5 μm. (B) Live epifluorescence microscopy with Pf3D7_1437000-mNeonGreen (green) and PM V-mRuby3 (red). Scale bars: 5 μm. White arrows are to give the reader spatial references between pictures. A representative image from cells cultured on three separate days is displayed here. Additional zoomed-out images are in Fig. S4B. (C) Quantification of signal overlap from B by Pearson's correlation coefficient. *n*=15 cells. Groups were compared by an unpaired two-tailed *t*-test, ****P<0.0001. Points represent the Pearson's correlation coefficient for individual cells quantified, error bars represent the s.d.

We assessed the localization of Pf3D7_1437000-HA in fixed parasites by immunofluorescence. Staining with anti-HA antibodies suggested that Pf3D7_1437000-HA is found in a perinuclear ring, consistent with ER localization (Fig. 3A) (Klemba et al., 2004). This was supported by co-staining with antibodies against organellar markers: anti-PM V for the ER (Banerjee et al., 2002; Klemba and Goldberg, 2005), anti-ACP for the apicoplast (Waller et al., 1998) and anti-aldolase for the cytosol. The staining pattern of anti-PM V largely resembled that of anti-HA, whereas anti-ACP and anti-aldolase clearly highlighted patterns distinct from that of anti-HA. To confirm that this localization is not an artifact of cell fixation or antibody staining, we constructed a parasite line with Pf3D7_1437000 fused to the fluorescent protein mNeonGreen and 3xHA, and PM V fused to mRuby3 and 3xFLAG. Western blotting showed most tagged protein to be at the expected size for full-length Pf3D7_1437000-mNeonGreen-3xHA and PM V-mRuby3-3xFLAG (Fig. S4). Live microscopy on trophozoites showed colocalization between Pf3D7_1437000-mNeonGreen and PM V-mRuby3 (Fig. 3B,C), again suggesting that Pf3D7_1437000 localizes to the parasite ER. Interestingly, we note that, both by immunofluorescence and live fluorescence, Pf3D7_1437000 and PM V appeared to occupy the same space, but their intensities were not perfectly correlated throughout that space: PM V was present in a ring around the nucleus with a single protruding bleb; Pf3D7_1437000 was present over the same space, but disproportionately concentrated in the bleb. The significance of this distinction is not obvious to us.

### Pf3D7_1437000 is not required for protein export

**Fig. 4.**
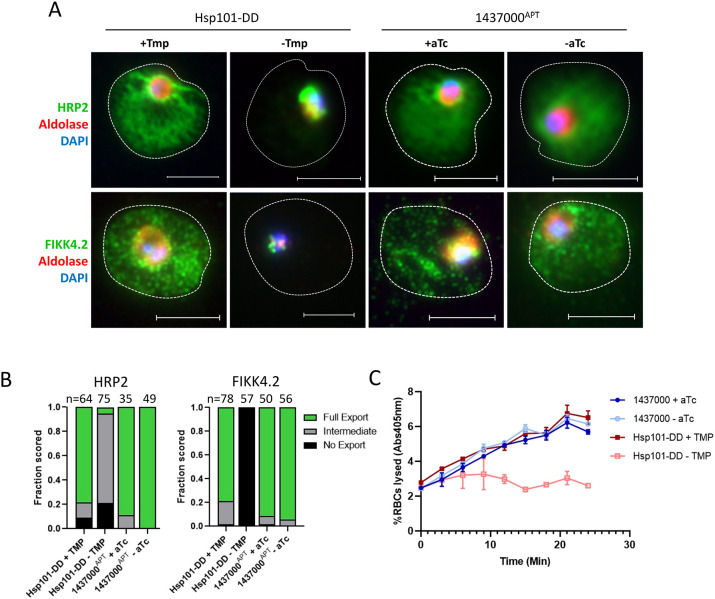
**Pf3D7-1437000 depletion does not affect protein export.** (A) Parasites were synchronized at the early ring stage, maintained in medium as shown with 10 μM trimethoprim (+TMP) or an equal volume of DMSO (−TMP), or 100 nM aTc (+aTc) or an equal volume of DMSO (−aTc). Parasites were fixed 24 h after invasion as trophozoites and stained to compare protein export in Hsp101-DD +/−TMP and 1437^APT^ +/−aTc. Exported proteins are marked by anti-HRP2 or anti-FIKK4.2, each shown in green; the parasite cytosol is marked by anti-aldolase, shown in red; parasite nucleus marked by DAPI is in blue. Experiment was performed three times; representative images are shown. Scale bars: 5 μm. (B) Quantification of ten fields by a scorer unaware of the identity of the samples for the experiment shown in A. Number of cells counted is shown above each bar. (C) Sorbitol sensitivity was monitored by measuring hemoglobin release into the supernatant (via absorbance at 405 nm) over time. Experiment was performed three times. A representative experiment is shown, with each sample in technical triplicate. Data points represent the measured mean, error bars the s.d.

Because our original interest was in the acetylation of exported proteins, we next sought to assess whether Pf3D7_1437000 has a role in exporting proteins into the host red blood cell (RBC). We used for comparison a previously described line in which the *Plasmodium* translocon for exported proteins (PTEX) component Hsp101 (PF3D7_1116800) is fused to a dihydrofolate reductase (DHFR) destabilization domain (abbreviated as Hsp101-DD), and its function requires the stabilizing ligand trimethoprim (TMP). When TMP is removed, Hsp101 is destabilized and exported proteins accumulate in the parasitophorous vacuole (Beck et al., 2014). Alongside this line, we depleted Pf3D7_1437000 by washing aTc from young ring-stage parasites (0–4 h old), then fixed trophozoites and stained for exported proteins by immunofluorescence assay (Fig. 4A). We found that although Hsp101 destabilization blocked protein export as previously described, depletion of Pf3D7_1437000 had no discernible effect on export of the PEXEL proteins HRP II or FIKK4.2 (Pf3D7_0424700) (Fig. 4A,B).

To get an immunofluorescence-independent view of export competence, we assessed establishment of the exported protein-dependent nutrient import channel on the infected erythrocyte surface (Beck et al., 2014; Ginsburg et al., 1985). To do so, we depleted Pf3D7_1437000 or disrupted Hsp101, pelleted trophozoites and resuspended them in 5% sorbitol. Export-competent parasites have increased solute uptake into the host RBC, leaving them susceptible to osmotic lysis in 5% sorbitol. Parasites that cannot export proteins are not able to increase solute uptake and are protected from sorbitol lysis (Beck et al., 2014; Ginsburg et al., 1985; Kirk et al., 1994). We monitored lysis by measuring the release of hemoglobin into the supernatant over 25 min. Disrupting Hsp101 protected parasites from lysis, as previously described (Beck et al., 2014), but depleting Pf3D7_1437000 had no effect on sorbitol sensitivity (Fig. 4C), again suggesting that Pf3D7_1437000 is not involved in protein export.

**Fig. 5.**
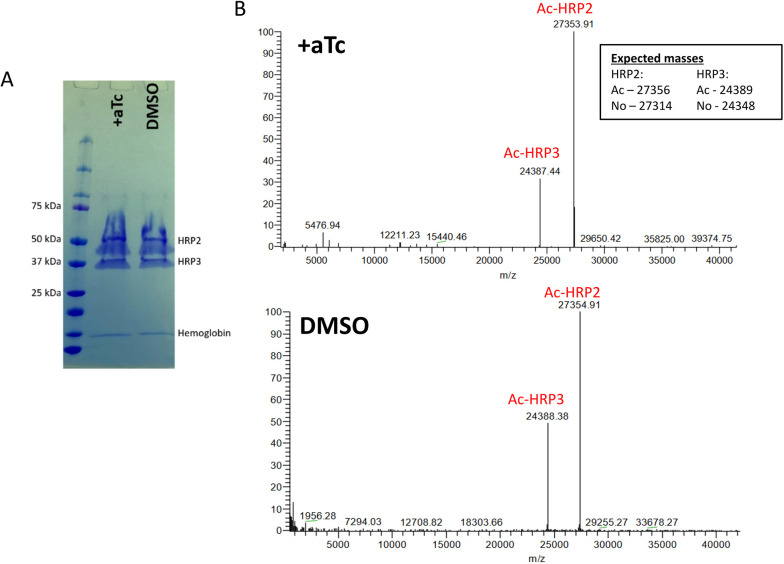
**Pf3D7_1437000 depletion does not affect HRP2 or HRP3 acetylation.** Schizont-stage parasites were washed to remove aTc, then lysed 48 h later, and HRP2 and HRP3 were purified by nickel-affinity chromatography. (A) Coomassie Brilliant Blue-stained SDS-PAGE gel showing HRP2 and HRP3 isolated from the supernatants of saponin-released parasites. Both migrate through the gel less than one would anticipate from their linear size due to their extreme positive charge. (B) Deconvoluted mass spectra from analysis of intact HRP2 and HRP3. Inset shows anticipated sizes for acetylated and unacetylated HRP2 and HRP3 after *Plasmodium* export element cleavage. This experiment was performed twice; a representative experiment is shown. Ac, acetylated; No, unacetylated.

To determine whether Pf3D7_1437000 is responsible for acetylating export-destined proteins in the ER, we isolated two abundant exported parasite proteins, histidine-rich proteins II and III [HRP2 (Pf3D7_0831800) and HRP3 (Pf3D7_1372200), respectively] (Fig. 5A) and measured the mass of each intact protein by liquid chromatography–mass spectrometry. In the presence of aTc, we detected substantial peaks consistent with acetylated HRP2 and acetylated HRP3, as expected (Fig. 5B). When we depleted Pf3D7_1437000, we were surprised to find no change in the mass of the peaks (Fig. 5B). In fact, the chromatogram in both cases revealed no peak at the mass expected for unacetylated HRP2 or HRP3.

**Fig. 6.**
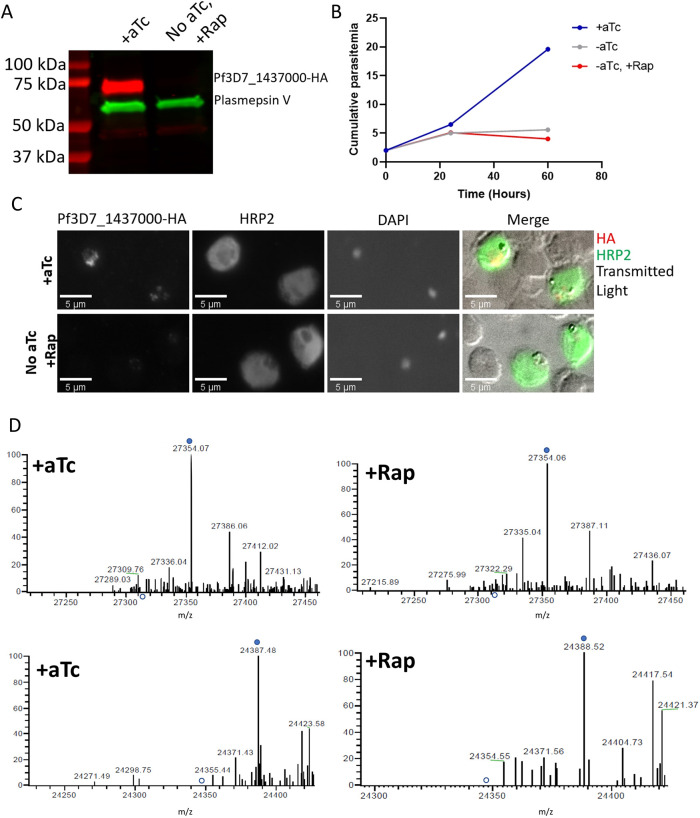
**More stringent dimerizable Cre recombinase-mediated Pf3D7_1437000 excision does not reveal a role for Pf3D7_1437000.** (A) Western blot comparing levels of Pf3D7_1437000 (red) and plasmepsin V (green) in parasites after 48 h treatment with aTc or washing out aTc and adding 50 nM rapamycin. (B) Parasites were cultured in the presence or absence of 50 nM rapamycin, and their growth was followed daily by flow cytometry. The experiment was performed three times, each time in technical triplicate. A representative experiment is shown. (C) Trophozoites were fixed and stained to compare levels of Pf3D7_1437000–HA and the exported protein HRP2 with aTc, or with aTc removed and rapamycin added. The experiment was performed two times; representative images are shown. (D) Zoomed-in portions of deconvoluted mass spectra from analysis of intact HRP2 (top row) and HRP3 (bottom row), isolated from saponin-released parasite lysates from aTc- or rapamycin-treated lines containing floxed Pf3d7_1437000-HA by nickel-affinity chromatography. Filled blue circles indicate the m/z expected for Ac-HRP2 and Ac-HRP3; open blue circles indicate expected m/z for the unacetylated HRP2 or HRP3. This experiment was performed twice; a representative experiment is shown. Full spectra and the gel are presented in Fig. S5.

Concerned that this could be due to the incomplete aptamer-driven knockdown despite the lethality phenotype, we retransfected the aptamer construct in a parasite line expressing a dimerizable Cre recombinase (DiCre) activated by the ligand rapamycin (Fierro et al., 2022 preprint; Jullien et al., 2003). Because our aptamer construct already resulted in a gene flanked by loxP sites (Fig. 2A), this allowed us to inducibly excise the Pf3D7_1437000 gene from the genome. Addition of 50 nM rapamycin to the culture medium of synchronized trophozoite-stage parasites for 48 h depleted Pf3D7_1437000-HA to undetectable levels (Fig. 6A) and resulted in parasites that were unable to grow (Fig. 6B). Despite near-complete Pf3D7_1437000 depletion, we again saw no difference in HRP2 export (Fig. 6C). We purified HRP2 and HRP3 from parasite culture and again found the mass of each unchanged by the excision of Pf3D7_1437000 from the genome (Fig. 6D; Fig. S5). To decrease the chance that we were merely failing to ionize/detect intact unacetylated HRP2 or HRP3, we also used endoproteinase Glu-C to proteolytically fragment HRP2 and HRP3, then used tandem mass spectrometry to identify fragments. All the N-terminal peptides that we detected for these two PEXEL proteins were acetylated, even in the rapamycin-treated parasites (Fig. S6, Table S2). This suggests that Pf3D7_1437000 is not the NAT that acetylates PEXEL proteins.

### Pf3D7_1437000 is involved in parasite growth and entry into schizogony

**Fig. 7.**
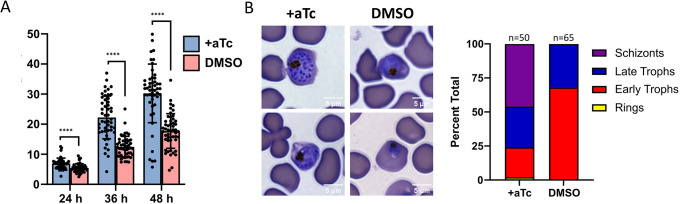
**Pf3D7_1437000 depletion results in growth arrest before schizogony.** (A) Area of parasites +/−aTc examined by thin smear at the indicated times post-infection. Parasites were synchronized at the early ring stage and maintained in medium with 100 nM aTc (+aTc) or an equal volume of DMSO (DMSO). Bar height indicates mean of 50 measured parasites, error bars indicate s.d. For each time point, the measured areas were compared by an unpaired two-tailed *t*-test, ****P<0.0001. (B) Left: demonstrative images showing phenotypes described in the text. +aTc parasites were mostly schizonts and late trophozoites; parasites without aTc arrested before schizogony. Scale bars: 5 μm. Right: quantification of parasite life stages of ten microscopic fields (*n*=50 or *n*=65 parasites, shown at the top of bars). Experiment was performed twice. A representative experiment is shown.

We next turned our attention to the role of Pf3D7_1437000 in intraerythrocytic development. Normally, parasites are classified as ring forms for the first 20–24 h after invasion, followed by maturation to fuller trophozoite forms and finally to schizonts that have replicated their DNA and formed daughter merozoites, the invasive forms that will invade new RBCs. We synchronized parasites within a 3-h window, maintained them with or without aTc, and monitored parasite development by thin smear. By 24 h after invasion, Pf3D7_1437000-depleted parasites appeared smaller than their non-depleted counterparts (Fig. 7A; mean area: +aTc, 6.95 μm^2^; DMSO, 5.45 μm^2^; unpaired two-tailed *t*-test, *P*<0.0001). The disparity in size persisted throughout the intraerythrocytic development cycle, with Pf3D7_1437000-depleted parasites approximately half the size of non-depleted parasites by 36 h (mean area: 22 μm^2^ versus 12 μm^2^) (Fig. 7A). By 48 h after invasion, non-depleted parasites had formed schizonts filled with clearly distinguishable daughter cells. Pf3D7_1437000-depleted parasites apparently remained trophozoites – some mature but without distinguishable daughter cells, others shrunken (Fig. 7B). Correspondingly Pf3D7_1437000-depleted parasites remained much smaller than non-depleted parasites (mean area: 18 μm^2^ versus 30 μm^2^) (Fig. 7A). Thus, we conclude that Pf3D7_1437000 is important for parasite growth and for completion of proper schizogony.

**Fig. 8.**
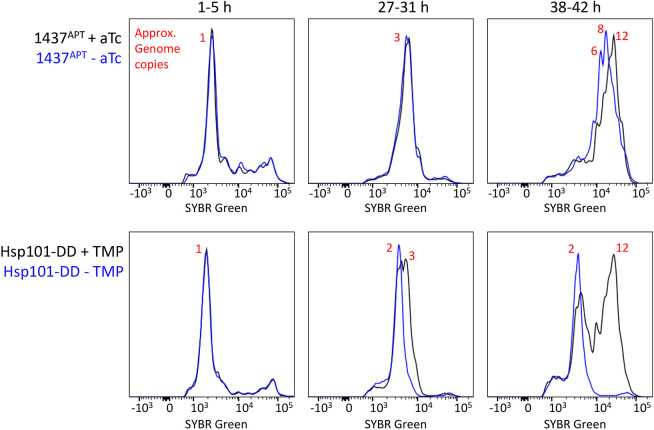
**Pf3D7_1437000-depleted parasites do not complete DNA replication.** Synchronized parasites were maintained as before, fixed at the indicated times, and their DNA content was measured with SYBR Green I and flow cytometry. Samples were run in technical triplicate (*n*=100,000 cells per sample). Uninfected red blood cells were gated out by their low SYBR Green I fluorescence. Histograms are shown, with SYBR Green I fluorescence along the *x*-axis and frequency on the *y*-axis. Approximate genome copy numbers were estimated from each peak's fluorescence intensity, assuming that most parasites at 1–5 h after invasion have a single copy of the genome. This experiment was performed twice; a representative experiment is shown.

The Pf3D7_1437000 knockdown phenotype appeared to us distinct from death due to protein export block, which arrests growth at the transition from rings to trophozoites (Beck et al., 2014). To distinguish these phenotypes more clearly, we fixed Pf3D7_1437000-regulated parasites alongside Hsp101-DD parasites at several time points during the intraerythrocytic development cycle and assessed DNA content with the dye SYBR Green I by flow cytometry (Fig. 8). By 28 h after invasion, Hsp101-disrupted parasites lagged behind non-disrupted parasites, whereas Pf3D7_1437000-depleted parasites were indistinguishable from their non-depleted partners. By 40 h after invasion, Hsp101-disrupted parasites had the same DNA content that they did 20 h earlier. Pf3D7_1437000-depleted parasites too lagged behind their non-depleted counterparts, but with a substantially larger DNA content. Thus, it appears that growth arrest caused by Pf3D7_1437000 depletion is distinct from and substantially later than arrest caused by export disruption. Disrupting export prevents most of S-phase, while Pf3D7_1437000-depleted parasites continue until almost the final rounds of DNA replication.

## DISCUSSION

Here, we sought to investigate the acetylation of PEXEL proteins, instead finding a putative NAT that is essential for parasite growth but appears uninvolved in protein export and is likely not the PEXEL NAT. Recent work using N-terminal proteomics found that a high percentage of N-termini detected from secretory-resident proteins were acetylated (six of 16 in *Plasmodium berghei*, 15 of 23 in *T. gondii*) (Nyonda et al., 2022), suggesting broad N-terminal acetylation in the apicomplexan secretory system. Pf3D7_1437000 seemed a likely candidate (and really the only plausible candidate) to accomplish this secretory acetylation. Indeed, there is explicit speculation in the literature that it might serve such a role (Boddey and Cowman, 2013; Nyonda et al., 2022; Osborne et al., 2010). Supporting that is its putative ER localization: it appears in Marapana et al.’s extensive immunoprecipitation and mass spectrometry results when pulling down SPC21 (signal peptidase) and SPC25 (Marapana et al., 2018). It has a clear *T. gondii* ortholog that has been localized to the ER by endogenous tagging and immunofluorescence (Nyonda et al., 2022), and tag-free proteomics (Barylyuk et al., 2020). Our findings are consistent with this ER localization, yet argue against Pf3D7_1437000 being the apicomplexan ER NAT.

Our data most parsimoniously support a model in which Pf3D7_1437000 is not the PEXEL NAT; however, our data cannot yet exclude alternative models. First, PF3D7_1437000 could be so efficient at acetylating its substrates that the small amount remaining after knockdown could acetylate all HRP2 and HRP3 N-termini that we could detect. We tried to minimize this possibility by harvesting schizonts for our mass spectrometry experiments, giving any acetylation phenotype as much time as possible to manifest. We also used two different depletion methods. At the time of harvest, Pf3D7_1437000 knockdown and knockout parasites have been physically affected by the depletion (as measured by parasite size) for at least 20 h, suggesting that the remaining enzyme is not able to accomplish its full function for much of the life cycle. Still, the possibility remains that Pf3D7_1437000 is acetylating HRP2 and HRP3 in our assay, and that parasite growth problems are caused by a distinct second function of this protein that is more sensitive to its levels. Second, it remains possible that Pf3D7_1437000 depletion affects HRP2 and/or HRP3 acetylation, but we failed to detect the unacetylated forms due to their poor stability in the parasites, solubility in our purification system or ionization for mass spectrometry. The only data we have to consider in this light is that Tarr et al. described mutants of the reporter fusion REX3^1-61^-GFP that are unacetylated (and not exported) and appear in similar abundance to that of the wild-type protein (Tarr et al., 2013). That is, acetylation of the PEXEL N-terminus is not universally required for protein stability and detection; however, we cannot exclude the possibility that such problems have frustrated our analysis here. A third possibility is that an alternative NAT acetylates HRP2 and/or HRP3 when Pf3D7_1437000 is depleted. In eukaryotic cells, stress can induce unconventional protein secretion pathways that allow ER or Golgi-independent secretion of proteins (Giuliani et al., 2011; Rabouille, 2017). As Pf3D7_1437000 depletion is undoubtedly stressful to the parasite and potentially to the ER, it is possible that, under such conditions, processed HRP2 and HRP3 are accessible to cytoplasmic NATs that acetylate them.

Regardless of whether Pf3D7_1437000 is the PEXEL NAT, we find that its depletion does not affect protein export, and that the phenotype of its depletion differs from described disruptions of export machinery. Depletions and disruptions of the major PTEX components Hsp101 and PTEX150 have been described, each of which arrest parasite growth as early trophozoites (Beck et al., 2014; Elsworth et al., 2014). Chemical inhibition or depletion of PM V can arrest parasite growth immediately after invasion, or as early trophozoites (Boonyalai et al., 2018; Polino et al., 2020; Sleebs et al., 2014). Here, we show that Pf3D7_1437000 depletion arrests parasite growth substantially later than blocking export via Hsp101 disruption. Our finding that Pf3D7_1437000 depletion affects parasite size through much of intraerythrocytic development cycle is curious, but its causes could be manifold, and further study of Pf3D7_1437000-depleted parasites would be needed to elucidate how this putative enzyme influences cell size. Our aptamer-mediated knockdown approach indicated that the parasite is highly sensitive to modest decrease in Pf3D7_1437000, as 60% reduction of the protein was lethal. However, it is also possible that the C-terminal tagging of the protein affects its function and localization. The ‘bleb’ formation could be a consequence of misfolding and accumulation in the ER and might explain why only 60% knockdown was lethal.

Among our field's directives is to identify and characterize essential parasite proteins and pathways sufficiently diverged from their hosts' orthologs as to be specifically targeted with chemotherapeutics. In some ways, Pf3D7_1437000 fits that bill: an essential putative NAT with orthologues throughout Apicomplexa, yet without a clear ortholog in mammals. However, hurdles remain before its druggability can be assessed. First, the degree to which this enzyme is essential in other pathogenic apicomplexans is not yet known. The *P. berghei* knockout screen scored the *P. berghei* ortholog PbANKA_0611800 as ‘essential’, albeit with low confidence (Bushell et al., 2017), while inducible depletion of the *T. gondii* ortholog TgME49_305450 had only a modest defect during repeated infection (i.e. plaquing) (Nyonda et al., 2022). Delineating how well conserved the function and essentiality of Pf3D7_1437000 is remains for future work. Perhaps more importantly – assuming Pf3D7_1437000's essential function is as an NAT – chemical inhibition of NATs has only been described with acetyl-CoA-peptide conjugates (Foyn et al., 2013). The development of more druglike and cell-permeant NAT inhibitors might raise the profile of this enzyme class for additional chemotherapeutic development.

Our data leave us with a question: if Pf3D7_1437000 is not the PEXEL NAT, what is? The simplest possibility is that another NAT resides in the parasite ER, with the most obvious candidates being the additional members of the GNAT superfamily listed in Fig. 1. Each has an ortholog with an alternative described function in yeast and mammals, but perhaps one serves a distinct role in Apicomplexa. An alternative is that the genome continues to hide an as-yet cryptic NAT, the sequence of which defies our attempts to computationally predict its function. Unfortunately, no obvious candidate jumps out from the Marapana et al. immunoprecipitations of various ER proteins involved in PEXEL processing (Marapana et al., 2018). A third possibility is that no protein NAT truly resides in the parasite ER, but instead that following PEXEL cleavage by PM V, the new N-terminus somehow accesses the cytosol and is acetylated by the regular cadre of ribosome-associated NATs. Tarr et al. used a split-GFP setup to show that the active site of PM V is oriented into the ER lumen (Tarr and Osborne, 2015), and we have since presumed that the post-cleavage PEXEL N-terminus is limited to the ER lumen. However, our understanding of the dynamics of secretory import and traffic in *Plasmodium* is limited, and alternative models have been proposed in which the neo-N-terminals of PEXEL proteins are exposed to cytosolic NATs (Römisch, 2012).

Lastly, the determinants of exported protein trafficking in *P. falciparum* remain unclear. Here, we were unable to assess the role of PEXEL acetylation on export competence. However, our mass spectrometry approach measured the mass of intact HRP2 and HRP3 and found each to be the exact mass expected from the polypeptide backbone and N-terminal acetylation alone, suggesting that export competent proteins need no further post-translational modification beyond what has already been described.

Taken together, our data provide new insight into the processing of exported proteins – if largely by excluding the primary candidate for involvement in the process. We provide initial characterization of the putative NAT Pf3D7_1437000, the essential function of which in schizogony remains unclear. At this moment, unbiased proteomics approaches, like comparing the levels of N-terminal acetylation or lysine acetylation of the total proteome, or comparing the ER proteome of *P. falciparum* between the wild type and Pf3D7_1437000 knockout mutant might be a good a way to shed light on the function of Pf3D7_1437000. It is also theoretically possible that PF3D7_1437000 N-terminally acetylates a subset of PEXEL proteins that does not include HRP2 and HRP3. We hope that future work will uncover the identity and role of the PEXEL NATs, and also clarify the role of Pf3D7_1437000 in the *Plasmodium* life cycle.

## MATERIALS AND METHODS

### Candidate NAT search

To search for candidate NATs, we used PlasmoDB (Amos et al., 2022) to generate a list of GNAT enzyme superfamily members. We manually checked the list, excluding candidates with a different predicted function (see text). Orthologs were identified based on grouping in OrthoMCL (Amos et al., 2022) and supported by reciprocal BLAST searches (NCBI; https://blast.ncbi.nlm.nih.gov/Blast.cgi). ER-targeting sequences were sought with SignalP 4.1 (Petersen et al., 2011), TMs were annotated based on TMHMM 2.0 predictions (Krogh et al., 2001). To predict membrane topology, we used TOPCONS (Tsirigos et al., 2015; https://topcons.cbr.su.se/pred/).

### Generation of plasmids

The construct for regulating Pf3D7_1437000 levels was made using the previously described pSN054 vector (Nasamu et al., 2021; Polino et al., 2020). Primer sequences are listed in Table S1; shorthand names will be used here. Homologous sequences for genome repair were amplified from NF54^attB^ (Nkrumah et al., 2006) with primers 14APT-1/14APT-2 for the left/upstream homologous region, and 14APT-3/14APT-4 for the right/downstream homologous region. The resulting PCR products were inserted into pSN054 cut with FseI and I-SceI, respectively, via Gibson Assembly (New England Biolabs). The recodonized Pf3D7_1437000 gene was synthesized by GENEWIZ (South Plainfield, NJ) and inserted into pSN054 containing the above homologous regions. Plasmid was cut with AsiSI, and the recodonized gene was inserted via Gibson Assembly to make the final vector called pSN054-1437000-3xHA. The vector was transformed into BigEasy-TSA Electrocompetent Cells (Lucigen) for propagation. When amplifying vector for harvest, 0.01% w/v arabinose was added to stimulate plasmid replication.

The two endogenous tagging vectors used for live microscopy – pM2GT-1437000-mNeonGreen-3xHA (yDHOD) and pM2GT-PMV-mRuby3-3xFLAG (hDHFR) – are derived from pM2GT-EXP2-mNeonGreen (yDHOD) (Glushakova et al., 2017) and pM2GT-Hsp101-3xFLAG (Garten et al., 2018; Ho et al., 2018). For the former, mNeonGreen-3xHA was amplified (and the HA added) by primers NG-HA-F/NG-HA-R. The amplicon was inserted into pM2GT-EXP2-mNeonGreen cut with AvrII/EagI via In-Fusion Cloning (Takara). For tagging Pf3D7_1437000, homologous sequences were amplified from the NF54^attB^ genome with primers 14NG-1/14NG-2 for the right homologous region (in the 3′ UTR) and 14NG-3/14NG-4 for the left homologous region. The two PCR products were combined in an overlap-extension PCR with the right homologous region forward and left homologous region reverse primers, and the resulting PCR product gel was extracted and inserted into the XhoI/NheI-cut plasmid via In-Fusion Cloning to make the donor vector pM2GT-1437000-mNeonGreen-3xHA, which was then transformed into XL10-Gold Ultracompetent Cells (Stratagene) for propagation.

Synthesis of the PM V tagging vector was analogous. First, mRuby3 was amplified with primers Rub-H-F/Rub-H-R and added to pM2GT-Hsp101-3xFLAG (hDHFR) (Garten et al., 2018; Ho et al., 2018) cut with AvrII using In-Fusion Cloning. To adapt the plasmid to PM V tagging, we used primers PMVR-1/PMVR-2 and PMVR-3/PMVR-4 to amplify the right and left homologous regions, respectively. These PCR products were inserted into the Xho/NheI-cut plasmid in a single pot reaction with In-Fusion Cloning, and the resulting vector was transformed into XL10-Gold cells for propagation.

CRIPSR/Cas9 targeting plasmids for each were made in the previously described pAIO3 plasmid (Nessel et al., 2020). Primers 14G-1, 14G-2, 14G-3, 14G-4, PMVG-1 and PMVG-2 were each ordered, along with their reverse complement sequences, annealed in the thermal cycler and then inserted into AvrII-cut pAIO3 by In-Fusion Cloning. Completed vectors were transformed into XL10 Gold cells (Agilent) for propagation.

### Parasite culture

For all experiments described here, we cultured *P. falciparum* in PRMI 1640 (Gibco) supplemented with 0.25% (w/v) Albumax I, 15 mg/l hypoxanthine, 110 mg/l sodium pyruvate, 1.19 g/l HEPES, 2.52 g/l sodium bicarbonate, 2 g/l glucose and 10 mg/l gentamycin, with human RBCs added to 2% hematocrit. Parasite cultures were maintained in sealed chambers under a gas mixture consisting of 5% O_2_, 5% CO_2_ and 90% N_2_ at 37°C. Deidentified RBCs were obtained from Barnes-Jewish Hospital blood bank (St Louis, MO), St. Louis Children's Hospital blood bank (St Louis, MO) and the American National Red Cross.

### Generation of parasite lines

All genetic modifications described here were done in the parasite line NF54^AttB^ (referred to as ‘NF54’ throughout) or, where noted, the modified version that expresses dimerizable Cre recombinase (Fierro et al., 2022 preprint; Nkrumah et al., 2006). For each transfection, donor vectors were linearized if necessary (pSN054 is already linear), and 50 μg each of donor vector and pAIO3 with relevant guide were combined, ethanol precipitated to ensure sterility and dissolved in 100 μl sterile water. At the time of transfection, the dissolved DNA was brought up to 400 μl in cytomix (120 mM KCl, 0.15 mM CaCl_2_, 2 mM EGTA, 5 mM MgCl_2_, 10 mM K_2_HPO_4_ and 25 mM HEPES adjusted to pH 7.6 with KOH; plasmid is solubilized more effectively in water than cytomix, so we typically allow DNA to dissolve in 100 μl water, then when dissolved add 100 μl 2× cytomix and 200 μl 1× cytomix to bring it up to transfection volume) and transfected into ∼5% young ring-stage parasites with a Bio-Rad Gene Pulser II (settings: 0.31 kV, 0.950 μF, capacitance set to ‘High Cap’, resistance on the Pulse Controller II set to ‘Infinite’). Successful transfectants were selected with the relevant drug beginning 24 h after transfection: Blasticidin S (2.5 μg/ml; Thermo Fisher Scientific) for the aptamer line, DSM-1 (2 μM; Asinex) and WR-99210 (5 nM; gift from D. Jacobus of Jacobus Pharmaceutical Co., Plainsboro Township, NJ) for the mNeonGreen- and mRuby3-tagged line. Medium was changed daily for the week following transfection, then thrice weekly until parasites could be visualized by thin smear, typically 2–4 weeks after transfection.

### Validation of lines

Proper integration of the pSN054-1437000-3xHA vector was confirmed by Southern blotting. Genomic DNA from the parent and edited lines was isolated (QIAamp DNA Blood Miniprep Kit), and 10 μg of each was digested with HinDIII, separated overnight on a 0.7% agarose gel and transferred to nylon (Nytran SuPerCharge TurboBlotter, 0.45 μm, GE Healthcare) overnight. The blot was then probed with the left homologous region (PCR product of 14APT-1/14APT-2) labelled with alkaline phosphatase (Amersham AlkPhos Direct Labeling Kit; GE Healthcare) in hybridization buffer (Amersham) at 55°C overnight, washed twice each in primary wash buffer (120 g/l urea, 1 g/l SDS, 100 ml/l 0.5 M sodium phosphate pH 7, 8.7 g/l NaCl, 2 g/l Amersham blocking reagent) and secondary wash buffer (6.05 g/l Tris base, 5.6 g/l NaCl, 2 ml/l 1 M MgCl_2_, pH 10), then detected with Amersham CDP-Star Detection Reagent (GE Healthcare) and exposed to blue autoradiography film (MidSci, BX810) overnight.

Additional validation by western blotting was done exactly as described in Polino et al. (2020) (section ‘Validation of PMV^APT^ line’).

### Assessment of Pf3D7_1437000 depletion

Except where otherwise noted, infection with the aTc-regulatable line (1437^APT^) was synchronized by purifying schizonts on LD columns (Miltenyi Biotech), eluting into fresh blood and medium lacking aTc and allowing parasites to invade for 3–4 h. Invasion was halted by replacing medium with 5% sorbitol, lysing any unegressed schizonts. We then washed in fresh medium one additional time for 5 min, to ensure that aTc was removed from the culture. These synchronized parasites were cultured in either the presence of 100 nM aTc (‘+aTc’ throughout) or an equal volume of DMSO (‘DMSO’ throughout). DiCre excision experiments were performed as above, but parasites were cultured in the presence of 100 nM aTc and either 50 nM rapamycin or an equal volume of DMSO.

### Western blotting

We performed western blotting as in Polino et al. (2020) with primary antibodies mouse anti-HA diluted 1:1000 (clone 16B12; Biolegend, 901501), rabbit anti-HA diluted 1:1000 (Sigma-Aldrich, H6908), rabbit anti-PfAldolase diluted 1:2000 (Abcam, ab207494; targets the protein with PlasmoDB accession PF3D7_1444800) and mouse anti-FLAG diluted 1:500 (Sigma-Aldrich, F1804), followed by secondary antibodies goat anti-mouse IRDye 800CW (Licor) and donkey anti-rabbit IRDye 680RD (Licor), both diluted 1:10,000. For Fig. 2C, parasites were harvested at the indicated times and Pf3D7_1437000-3xHA levels were quantified using ImageStudio Lite v. 5.2 (Licor). The sizes of bands were approximated using the Precision Plus Protein Dual Color Standards (Bio-Rad, 1610374).

### Flow cytometery

To assess the effect of Pf3D7_1437000 depletion on parasite growth, parasites were maintained in technical triplicate (3×1 ml culture), and their growth was monitored daily by flow cytometry (BD FACSCanto with attached High Throughput Sampler) by diluting culture 1:20 into PBS with 0.8 μg/ml Acridine Orange (Molecular Probes).

We assessed parasite progress through the DNA replication cycle as in Perrin et al. (2021). Briefly, parasites were synchronized as above, then fixed at the indicated times (see Fig. 7) by doubling their volume in 2× PBS+0.4% glutaraldehyde (final concentration, 1× PBS+0.2% glutaraldehyde) and stored at 4°C until all time points had been collected. Then, DNA was stained using SYBR Green I (Thermo Fisher Scientific), measured on a BD FACSCanto (BD Biosciences) and analyzed with FloJo v.10.7.1 (BD Biosciences).

### Microscopy

For localization of Pf3D7_1437000, parasites were synchronized as above to within a 4-h window, then harvested 24 h after invasion ended (i.e. when parasites were 24–28 h old), washed once in PBS and prepared for immunofluorescence imaging as recommended in Tonkin et al. (2004), with the modification that cells were settled onto concanavalin A-coated coverslips (0.5 mg/ml) for 10 min prior to fixation, and that the wash following primary and secondary antibody incubation consisted of five PBS washes for 3 min each. Primary antibodies used were as follows: mouse anti-HA (close 16B12; Biolegend, 901501) diluted 1:100, rabbit anti-HA (Sigma-Aldrich, H6908) diluted 1:100, mouse anti-PMV (Banerjee et al., 2002) diluted 1:50, rabbit anti-ACP (Waller et al., 1998) diluted 1:100, and rabbit anti-aldolase (Abcam, ab207494) diluted 1:500. Secondary antibodies used were as follows: goat anti-mouse IgG-AlexaFluor488, goat anti-rabbit IgG-AlexaFluor555 (both from Invitrogen) diluted 1:2000. Coverslips were mounted in ProLong Gold Antifade with DAPI (Thermo Fisher Scientific), allowed to cure for 24 h, then imaged on a Zeiss AxioImager.M1 epifluorescence microscope with a Hamamatsu ORCA-ER CCD camera and AxioVision v. 4.8.1. Images were cropped, scale bars were added, and brightness and contrast were adjusted for presentation with Zen Lite v. 2.5 (Zeiss).

Parasites for live microscopy were synchronized as in the preceding paragraph, harvested 24 h after invasion, washed once in PBS, incubated with 1 μg/μl Hoechst 33342 (Invitrogen, H3570) for 30 min, then imaged on the same AxioImager.M1 described above. Images were analyzed in ImageJ (https://imagej.net/ij/): regions-of-interest were drawn around the mNeonGreen signal by hand, applied to all channels, and Pearson's correlation coefficients were calculated using ImageJ's coloc.pearsons() function.

To monitor parasite size, 1437^APT^ was synchronized as above and monitored by thin smear at the indicated times (see Fig. 6). Thin smears were fixed and stained with Harleco Hemacolor (MilliporeSigma), then imaged using a Zeiss Axio Observer.D1 at the Washington University Molecular Microbiology Imaging Facility. Parasite size was assessed using ImageJ, by manually drawing parasite borders and calculating their area.

To assess the effect of Pf3D7_1437000 depletion on protein export, 1437^APT^ was synchronized as above alongside Hsp101-DD (Beck et al., 2014), with Hsp101-DD maintained in 10 μM TMP or an equal volume of DMSO. Parasites were harvested 28 h after the invasion ended (i.e. parasites were 28–32 h old), fixed and processed as above. Primary antibodies were mouse anti-HRP2 clone 2G12 (Rock et al., 1987) diluted 1:500, mouse anti-FIKK4.2 (Kats et al., 2014) diluted 1:500, and mouse anti-KARHP clone 18.2 diluted 1:500. A scorer (K.F.) who was unaware of the identity of the samples scored five images per condition (number of parasites shown in Fig. 4B) as ‘full export’, ‘no export’ or ‘intermediate’.

### Sorbitol lysis assay

To measure sensitivity to sorbitol lysis, parasites were maintained as in the preceding paragraph. Twenty-four hours after invasion, parasites were moved to a 96-well plate (100 μl/well) and pelleted, and the medium supernatant was aspirated off. Every 5 min, we resuspended another row of the plate (four conditions, three technical replicates per condition) in 5% sorbitol. One row was instead resuspended in deionized water to fully lyse the RBCs. After 24 min, the infected RBCs were again pelleted, and the supernatant was transferred to a new 96-well plate. Lysis of infected RBCs was measured by absorbance at 405 nm, a measure of hemoglobin abundance, using an Envision Multimodal Plate Reader (PerkinElmer). Values are expressed relative to the deionized water control (representing 100% lysis). The cultures were at 6% parasitemia at the outset of the experiment, so a maximum expected value is 6% lysis.

### Assessment of HRP2/3 acetylation

To investigate the acetylation status of HRP2 and HRP3, we synchronized 1437APT parasites and maintained them as above. Forty-four hours after invasion, aTc was washed from cultures (three washes, 5 min each), and cultures were resuspended in 100 nM aTc or an equal volume of DMSO. For the DiCre experiments, either 50 nM rapamycin or an equivalent volume of DMSO was added. At the end of the following cycle (again, 44 h after invasion) parasites were pelleted, medium was aspirated, and then the RBCs and parasitophorous vacuole were lysed in 0.035% saponin in PBS at 4°C for 15 min. Parasite material was then pelleted at 17,000 ***g***, and the supernatant was poured onto nickel HTC agarose beads (Gold Biotechnology) that had been equilibrated in PBS+100 mM imidazole. Columns were washed in 20 column volumes of PBS+100 mM imidazole to remove the abundant hemoglobin, then eluted in 15 ml PBS+1 M imidazole. Eluate was concentrated in 10 K cutoff Amicon Ultra-15 Centrifugal Filters (Millipore), flash frozen in liquid nitrogen, then sent to the Danforth Plant Science Cetnter's Proteomics and Mass Spectrometry Facility. There, samples were acidified by formic acid to 1% then cleaned up with C4 ZipTip (Millipore). The captured samples were eluted with 50% acetonitrile in 0.1% formic acid, dried down and then resuspended in 10 μl 3% acetonitrile in 1% formic acid. Five microliters of sample were analyzed by liquid chromatography–mass spectrometry with a Dionex RSLCnano HPLC coupled to an OrbiTrap Fusion Lumos (Thermo Fisher Scientific) mass spectrometer using a 60 min gradient (2–90% acetonitrile). Sample was resolved using a 75 μm×150 cm PepMap C4 column (Thermo Fisher Scientific). Mass spectrometry spectra of protein ions of different charge states were acquired in positive ion mode with a resolution setting of 120,000 (at 200 m/z), and accurate mass was deconvoluted using Xcalibur (Thermo Fisher Scientific).

For the Glu-C digestion followed by mass spectrometry shown in Fig. S5, parasites were synchronized as above. Twenty-four hours after invasion, parasites were washed three times (5 min each) to remove aTc, and 50 nM rapamycin or an equal volume of DMSO was added. Forty-eight hours later, parasites were lysed, and HRP2 and HRP3 were purified as above. Samples were run on an SDS-PAGE gel, stained with Coomassie Brilliant Blue, and bands for HRP2 and HRP3 were cut and sent to the Mass Spectrometry Technology Access Center for further processing. There, gel bands were washed in 100 mM ammonium bicarbonate (AmBic)/acetonitrile and reduced with 10 mM dithiothreitol at 50°C for 30 min. Cysteines were alkylated with 100 mM iodoacetamide in the dark for 30 min in room temperature. Gel bands were washed in 100 mM AmBic/acetonitrile prior to adding 600 ng Glu-C for overnight incubation at 37°C. Supernatant containing peptides was saved into a new tube. Gel was washed at room temperature for 10 min with gentle shaking in 50% acetonitrile/5% formic acid, and supernatant was saved to peptide solution. The wash step was repeated each with 80% acetonitrile/5% formic acid, and 100% acetonitrile, and all supernatant was saved then subjected to the speedvac to dry. After lyophilization, peptides were reconstituted with 0.1% formic acid in water and injected onto a Neo trap cartridge coupled with an analytical column (75 µm ID×15 cm PepMap^TM^ RSLC C18, 3 µm). Samples were separated using a linear gradient of solvent A (0.1% formic acid in water) and solvent B (0.1% formic acid in acetonitrile) over 120 min using a Vanquish Neo UHPLC System coupled to an Orbitrap Eclipse Tribrid Mass Spectrometer (Thermo Fisher Scientific). Data were searched using Mascot (v.2.8.0 Matrix Science, Boston, MA) against a custom *Plasmodium falciparum* 3D7 database, and results were reported at 1% false discovery rate in Scaffold (v.5.1.1; Proteome Software, Portland, OR).

## Supplementary Material

10.1242/joces.260551_sup1 Supplementary information
