# Supplementary material for: An essential endoplasmic reticulum-resident N-acetyltransferase ortholog in Plasmodium falciparum
Source: J Cell Sci. 2023 Mar 6;136(6):jcs260551. doi: 10.1242/jcs.260551 (PMC10038149; doi:10.1242/jcs.260551)
Supplement: Supplementary information [file joces-136-260551-s1.pdf]

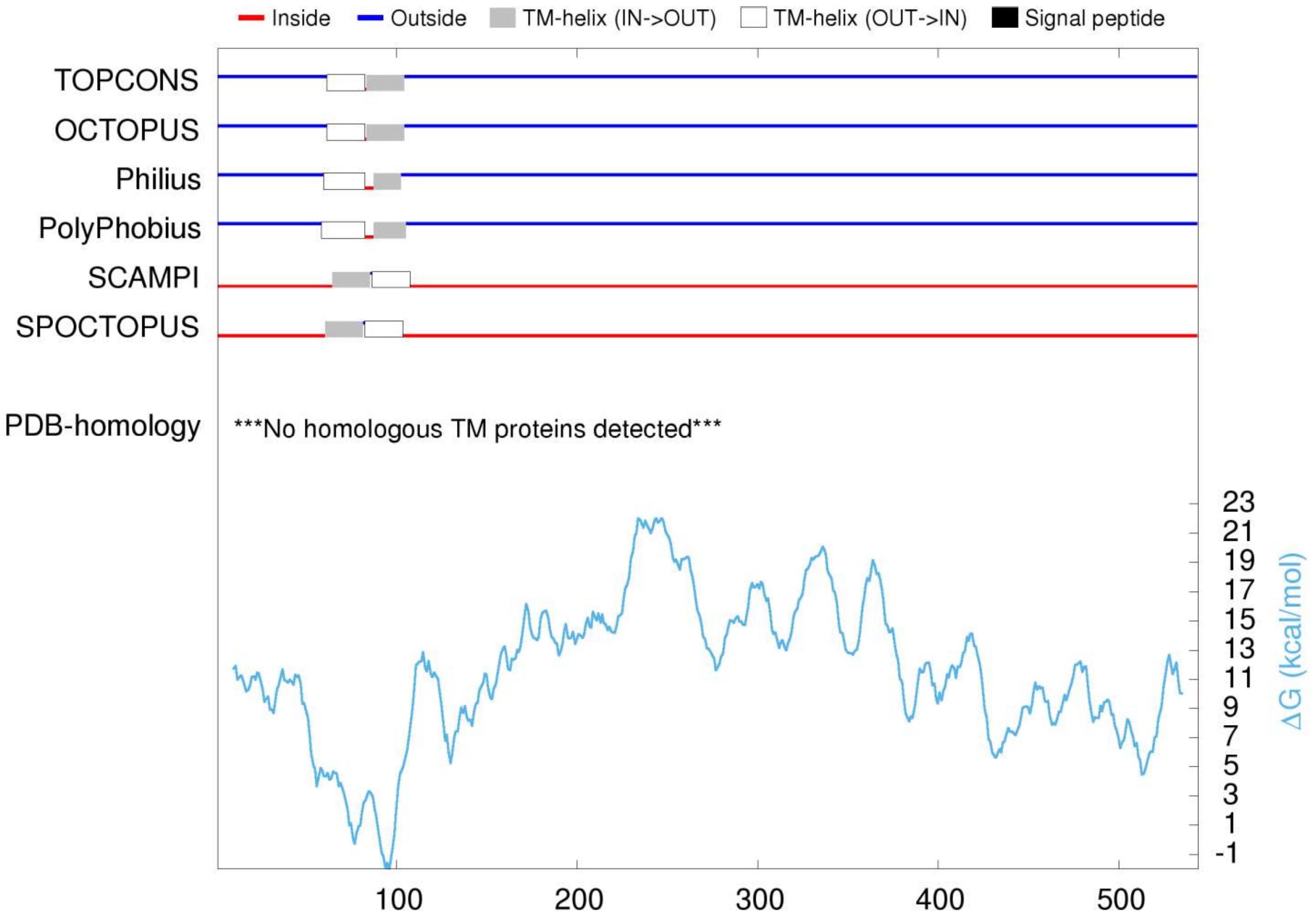

Blue: ER-lumen side
Red: Cytoplasmic side
Clear box: TM-helix ER lumen to cytoplasm
Grey box: TM-helix cytoplasm to ER lumen

Fig. S1. Membrane topology prediction for Pf3D7_1437000: TOPCONS web server predicts the membrane topology from a primary sequence. Topology is first predicted using five different algorithoms (rows 2 to 6) and then using them as inputs for a consensus prediction (top row). Note that the color keys provided by the server (shown above the first row) are for a cell membrane protein, which is reinterpreted for our purpose assuming Pf3D7_1437000 is an ER membrane protein (shown at the bottom)

Journal of Cell Science • Supplementary information

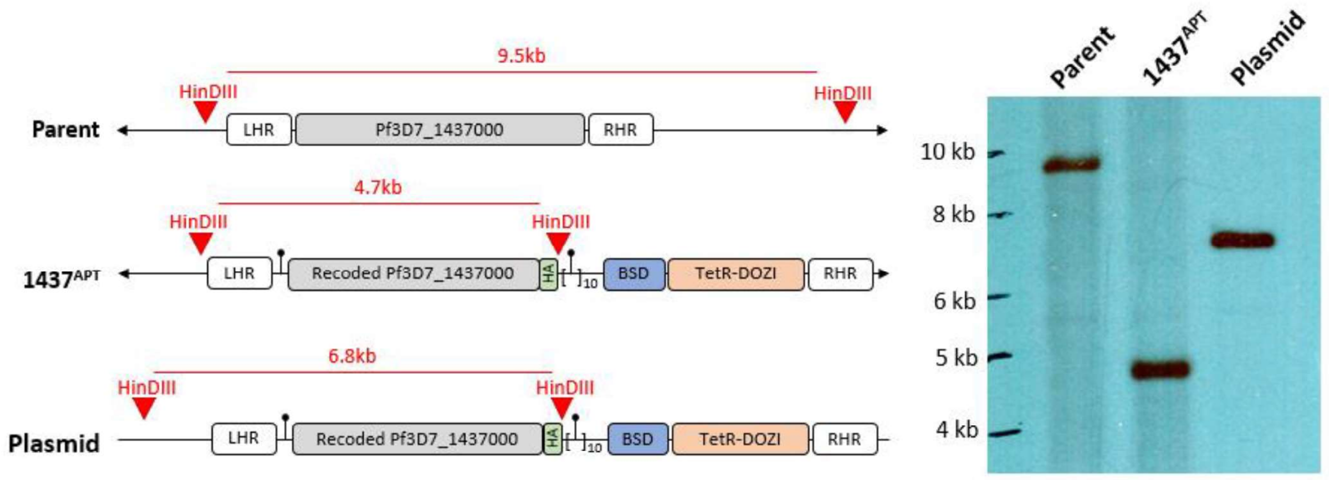

Fig S2. Southern blot: Correct genome editing was verified by Southern blot with left homologous region (LHR) used to probe the HinDIII-fragmented genome. Digest schematic shows expected size of bands for the parent, edited line (1437^{APT}) and donor plasmid.

Fig. 2, panel C:

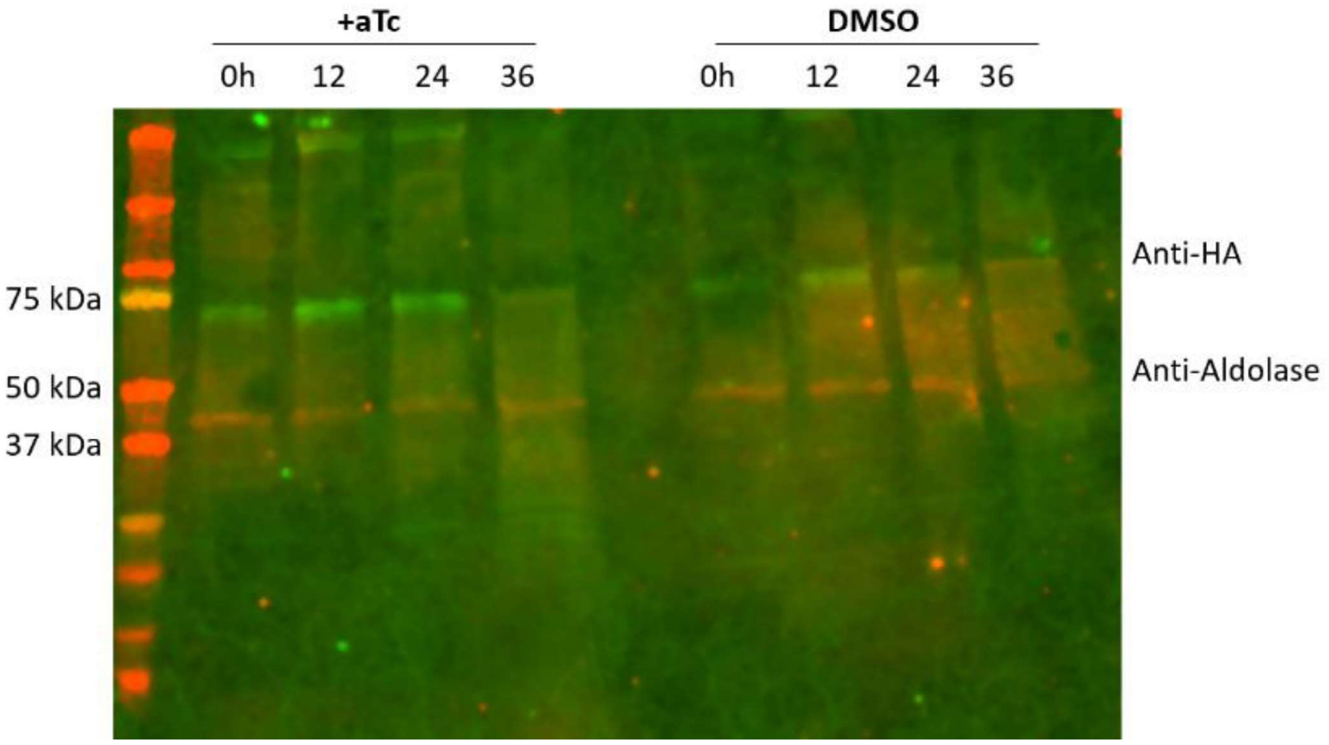

Fig. S3. Uncut blot for Figure 2

Journal of Cell Science • Supplementary information

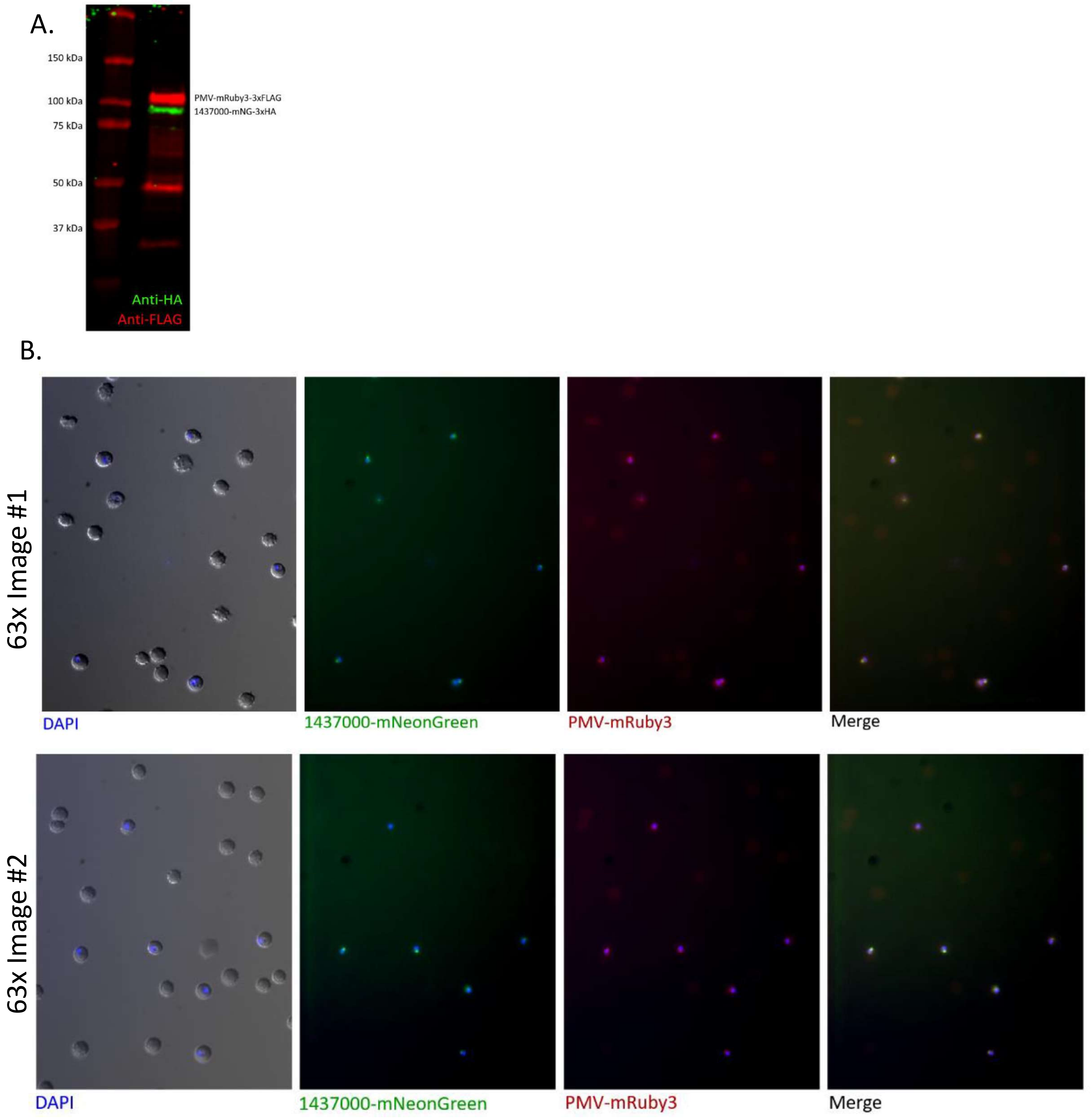

Fig. S4. Supplement to Fig. 3B. (A) Western blot probing Pf3D7_1437000-mNeonGreen-3xHA and PM VmRuby3-3xFLAG double-tagged line with antibodies for anti-HA (green) and anti-FLAG (red). **(B)** Additional images from Fig. 3B.

Journal of Cell Science • Supplementary information

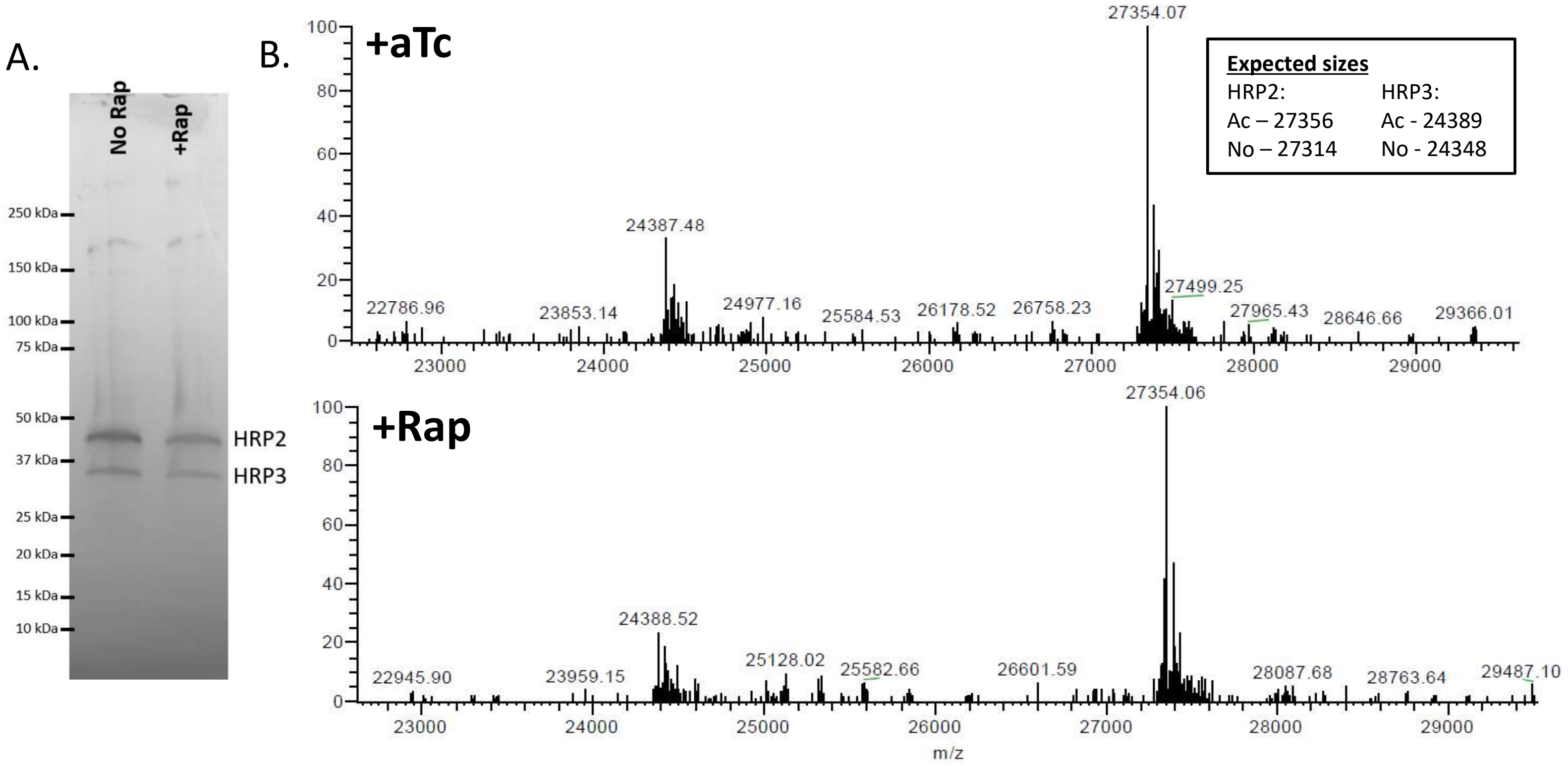

Fig. S5. DiCre excision of Pf3D7_1437000 locus also doesn't affect HRP2/HRP3 acetylation. 50nM rapamycin was added to schizont-stage parasites. 48 hours later, parasites were lysed and HRP2/HRP3 nickel affinity-purified as in Fig. 5. (A) Coomassie-stained gel showing results of HRP2/HRP3 purification from parasites treated with 50nM rapamycin (+Rap) or aTc (+aTc). (B) Deconvoluted mass spectra from analysis of intact HRP2 and HRP3. Inset shows anticipated sizes for acetylated and un-acetylated HRP2 and HRP3 after PEXEL cleavage.

Journal of Cell Science • Supplementary information

A

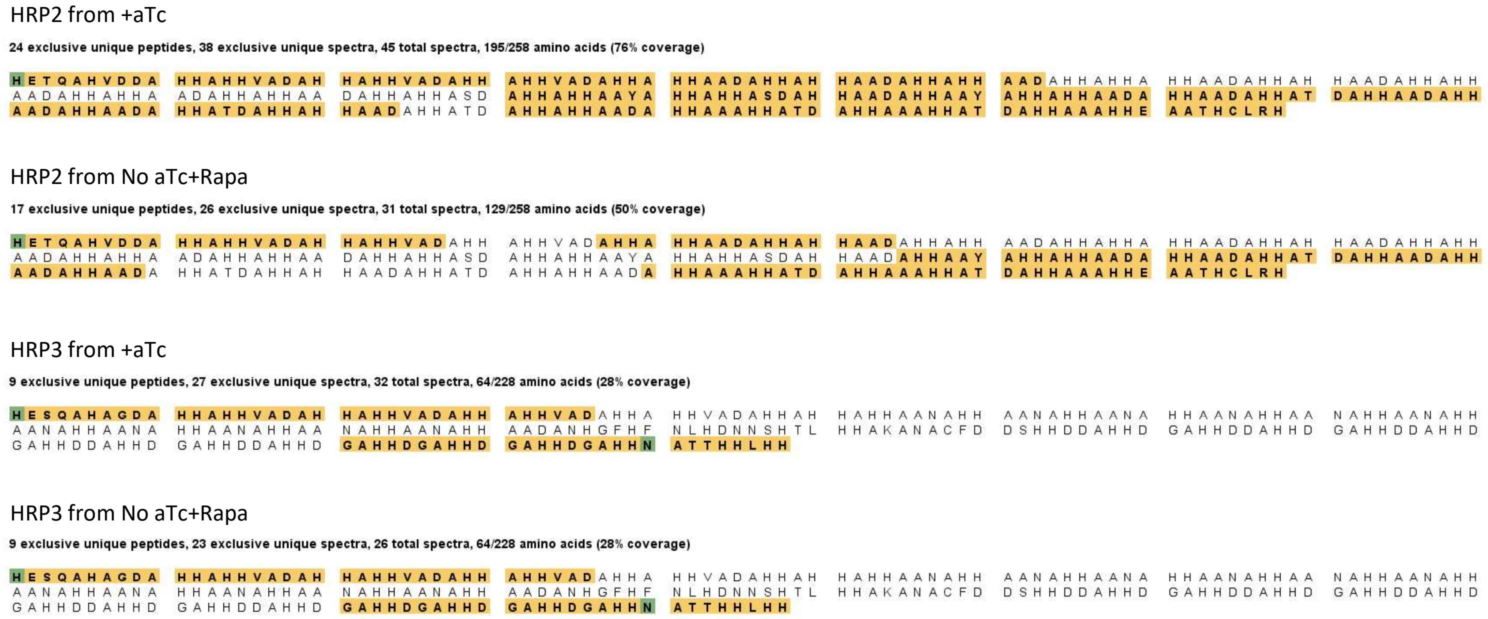

B

	HRP2		HRP3	
	+aTc	No aTc, +Rap	+aTc	No aTc, +Rap
#Acetylated N-term. peptides	4	3	4	5
#Unacetylated N-term. peptides	0	0	0	0
Total peptide spectra detected	45	31	32	26

Fig. S6. Only acetylated N-terminal peptides are detected for HRP2 and HRP3 proteins from Pf3D7_1437000 depleted parsites. HRP2 and HRP3 were isolated from saponin-released parasite lysates by nickelaffinity chromatography then digested with endoproteinase Glu-C and subjected to LC-MS/MS. (A) Peptide coverage map for HRP2 and HRP3 from the labeled samples detected from Mass spectrometry. Yellow shades corresponds to detected peptide sequences and green shade indicates post-translational modifications. (B) A summary table highlighting the number of acetylated and unacetylated N terminal peptides detected from mass spectrometry.

Journal of Cell Science • Supplementary information

Table S1. Primers used in this study

Name	Sequence
14APT-1	caaacttcattgactgtgccggccggccGAAGATTTTTCAATTGTTGATGGATGTGG
14APT-2	atgagctccggcaaatgacaagCCTTTTTGAAATCCATTTATACATCCATAAAAAAAATG
14APT-3	aacccggaattcgagctcggCCGTATTAATATTTATTTAATTTTAATGAAAAAAAATGTC
14APT-4	cgagagattgggtattagaccCATAATTTAAAAAAGATAGAAAAGAAGCACG
14NG-1	tgacactatagaactcgagGTATATATAATGATAAAAGGAATcGTCaC
14NG-2	cacatcctacttcgaaGATATACATGAAGAAACACACCCTCA
14NG-3	CTTCATGTATATCttcgaaGTAGGATGTGTAGGTATAGTACCG
14NG-4	ctccacttcccctaggATCtAgGATATTATTTGTATAGCTCTGTATTTCTG
PMVR-1	ggtgacactatagaactcgagCCCAATGCGCACATTTTATACTTACATC
PMVR-2	gtactacctgaatctacttcgaaCACATTTTGGCTAGTTTGTCCATGAATAATAAAAG
PMVR-3	ctagccaaaatgtgttcgaaGTAGATTCAGGTAGTACATTTACACATATTCCAG
PMVR-4	CtcctccacttccgctagcTGTTGATTCCTGTATGGGAGATTTATTTTGATCAgaCAgAGGAAAATAATCC
14G-1	taagtatataatatt**GAGCTATACAAATAATATCT**gttttagagctagaa
14G-2	taagtatataatatt**TTCATACATTTTTAATATTT**gttttagagctagaa
14G-3	taagtatataatatt**AAATTAAATAAATATTAATA**gttttagagctagaa
14G-4	taagtatataatatt**AAAGTCTTTAAAATATAAAT**gttttagagctagaa
PMVG-1	taagtatataatatt**AAATGTGCGCATTGGGAATG**gttttagagctagaa
PMVG-2	taagtatataatatt**GATTTATTTTGATCACTCAA**gttttagagctagaa
NG-HA-F	gatgaaaataaagaagctagcGGAAGTGGAGGAGTGAGCAAGGGCGAGGAGGATAA
NG-HA-R	TtatataactcgacgcggccgtcaGGCATAATCTGGAACATCGTAAGGATACGCATAATCGGGCACATCATAGGG ATAGCCAGCGTAGTCCGGGACGTCGTACGGGTAcctaggcttgtacagctcgtccatgcc
Rub-H-F	acttatctaagaccgctagcggaagtggaggaGTGTCTAAGGGCG
Rub-H-R	cgtccttgtagtccctaggCTTGTACAGCTCGTCCATGCCACC

Notes:
*Lowercase letters are overhanging sequence homologous to the vector backbone for In-Fusion or Gibson cloning
*Letters highlighted in yellow are shield mutations to avoid targeting the donor vector with CRISPR guides.
*Bolded sequences represent the CRISPR guide sequences inserted into pAIO3
*Sequence highlighted in red is 3xHA (or rather its reverse complement)

Journal of Cell Science • Supplementary information

Table S2. a multi-tab excel file listing all the proteins detected in bottom-up proteomics identification of HRP2 and HRP3 along with their identification probability and number of detected spectra. Peptideds detected from HRP2 and HRP3 are also listed on separate tabs.

Click here to download Table S2

Journal of Cell Science • Supplementary information
