# Supplementary material for: An essential endoplasmic reticulum-resident N-acetyltransferase ortholog in Plasmodium falciparum
Source: J Cell Sci. 2023 Mar 6;136(6):jcs260551. doi: 10.1242/jcs.260551 (PMC10038149; doi:10.1242/jcs.260551)
Supplement: Supplementary file 1 [file joces-136-260551_review_history.pdf]

An essential endoplasmic reticulum-resident N-acetyltransferase ortholog in *Plasmodium falciparum*

Alexander J. Polino, Muhammad M. Hasan, Katherine Floyd, Yolotzin Avila-Cruz, Yujuan Yang and Daniel E. Goldberg
DOI: 10.1242/jcs.260551

Editor: Michael Way

Review timeline

Original submission:	19 August 2022
Editorial decision:	11 October 2022
First revision received:	29 November 2022
Editorial decision:	19 January 2023
Second revision received:	24 January 2023
Accepted:	27 January 2023

Original submission

First decision letter

MS ID#: JOCES/2022/260551

MS TITLE: An essential ER-resident N-acetyltransferase in *Plasmodium falciparum*

AUTHORS: Alexander J Polino, Muhammad M Hasan, Katherine Floyd, Yolotzin Avila-Cruz, Yujuan Yang, and Daniel E. Goldberg
ARTICLE TYPE: Research Article

We have now reached a decision on the above manuscript.

To see the reviewers' reports and a copy of this decision letter, please go to: https://submit-jcs.biologists.org and click on the 'Manuscripts with Decisions' queue in the Author Area. (Corresponding author only has access to reviews.)

As you will see, the paper received very mixed reviews with the reviewers raising a number of substantial criticisms that prevent me from accepting the paper at this stage. They suggest, however, that a revised version might prove acceptable, if you can address their concerns. If you think that you can deal satisfactorily with the criticisms on revision, I would be pleased to see a revised manuscript. We would then return it to the reviewers.

Please ensure that you clearly highlight all changes made in the revised manuscript. Please avoid using 'Tracked changes' in Word files as these are lost in PDF conversion.

I should be grateful if you would also provide a point-by-point response detailing how you have dealt with the points raised by the reviewers in the 'Response to Reviewers' box. Please attend to all of the reviewers' comments. If you do not agree with any of their criticisms or suggestions please explain clearly why this is so.

Reviewer 1

Advance summary and potential significance to field

© 2023. Published by The Company of Biologists under the terms of the Creative Commons Attribution License (https://creativecommons.org/licenses/by/4.0/).

In this work Polino et al address the timely and important question of how proteins exported from Plasmodium falciparum into the red blood cell using the PEXEL targeting signal are N-acetylated. Mining the P. falciparum genome they identify 8 acetyl transferase homologs none of which contain an N-terminal signal sequence which would target them to the secretory pathway. One of them, however, contains two transmembrane domains which can also serve as ER-targeting signals. The authors therefore focus on characterizing this membrane-anchored N-acetyl transferase. They show that that the protein is ER-localized and essential, but its depletion does not affect N-acetylation of two PEXEL proteins, nor does it affect protein export from P. falciparum into the red blood cell. In addition, the death phenotype of depletion of this N-acetyl transferase differs from that of inhibiting export of PEXEL proteins. The authors conclude that the enzyme they investigated here is not the PEXEL N-acetylase, and offer a number of plausible explanations for their observations in their thoughtful discussion.

This paper is interesting, addresses a long-standing question in the malaria field, is very well written, and provides a balanced overview over the literature relevant to this investigation. The data presented is of high quality and largely supports the authors' conclusions.

Comments for the author

Nevertheless I have two major suggestions:

1. The charge distribution flanking the transmembrane domains determined the orientation of proteins targeted to the ER by transmembrane helices. There are a number of freely available prediction programmes that use this parameter to predict the topology of membrane spanning proteins in the ER. The authors should include a topology prediction of the Pf3D7_1437000 protein to see whether the NAT domain is even located in the lumen of the ER.

2. With the respect to the 'bleb' localization of the Pf3D7_1437000 protein this might indicate that a major fraction of the protein is misfolded in the parasites. What do the authors know about expression levels of the untagged protein? Is their construct significantly overexpressed? Or is it possible that any of the modifications introduced affect folding? If the majority of the expressed protein were misfolded and therefore non-functional, this might explain why the relatively moderate depletion down to 40% is already lethal. The authors need to address this issue.

Minor comments:

- page numbers are missing
- n numbers in Fig. 4B are too small to read
- both in the abstract and on the first page of the introduction the authors say PEXEL proteins are 'translated into the ER'. This has not formally been proven. In fact, proteins in which the PEXEL signal was reduced by site-directed mutagesesis to the plasmepsin V-cleaved form and preceded by a conventional ER targeting signal sequence led to the proteins accumulating in the parasitophorous vacuole, not in the red blood cell; so while PEXEL proteins are targeted to and translated at the ER membrane, whether they are actually translocated into the ER lumen remains unclear. It would be better to rephrase these sentences.

Reviewer 2

Advance summary and potential significance to field

The manuscript by Polino et al. reports the identification and characterization of NAT candidate in P. falciparum Pf3D7_1437000. The authors show that this protein is mainly localized in the ER and not required for protein export. However, downregulation of Pf3D7_1437000 prevents parasite development within the host red blood cell and affects the parasite's final DNA replication cycle. Most of these results are in line with the recent characterization of the homologous protein in T. gondii (TgNAT8) (Nyonda et al. 2022).
Overall, this is a nice and clear manuscript that contributes to an ER resident NAT's assessment of several biological functions of the parasite.

© 2023. Published by The Company of Biologists under the terms of the Creative Commons Attribution License (https://creativecommons.org/licenses/by/4.0/).

Most of the presented experiments appear accurately performed and well-presented, but the manuscript shows some limitations.

Major concerns

1) The most significant limitation of the study is the lack of N-terminomics data. We understand that specific pipelines are needed (available however) for the enrichment of N-termini and quantification of the N-terminal acetylation, but the conclusion cannot come from the presented mass spectrometry data. I do not suggest to purify ER prior of MS, but a global Nterminomics analysis must be presented for WT and KD samples.
2) The presented MS data is the weakest part of the manuscript and their presentation needs to be improved to meet the mass spectrometry standards. For instance, all MS spectra need to be deposited in a public repository (eg., PRIDE) and give the access number. Furthermore, it is necessary to add a complete excel table with the recovered peptides (including the few N-terms), position, localization, protein accession, N-terminal acetylation yield.. Figure 6D is useless except to point out the very low number of retrieved N-termini which cannot allow any conclusions to be drawn. This limitation need to be better explained in the result section and avoid any overstatement (e.g., this suggests that Pf3D7_1437000 is not the NAT that acetylates PEXEL proteins ect). All the hypotheses proposed in the discussion appear valid. However, without more experiments, the argument that Pf3D7_1437000 is not relevant for ER acetylation is purely speculative and unproven.

Minor comments

1) The introduction needs to be updated and it is regrettable that the authors do not cite and introduce already in this section recent advances in the field, making a fast conclusion that the identity of PEXEL remains to be determined.
2) I got lost where Supp. Fig. 2 is reporte in the text.
3) As for the NAT localization experiments, I'm a little confused about the claimed results. I can see the location of the ER, despite the unclear merging with the PMV stain. However, I see some of them merge with the localization of aldolase (cytosol). Please clarify the author's conclusion of this part. I would also suggest mentioning the localization of TgNAT8 from the recent Nyonda et al. 2022, in line with an ER localization.
4) Among the different explanations in the discussion, the authors do not consider the possibility that N-terminal acetylation of secretory proteins may occur after ER transit and cleavage of N-terminal PEXEL along the entire secretory pathway.

Comments for the author

Polino et al.
An essential ER-resident N-acetyltransferase in Plasmodium falciparum

The manuscript by Polino et al. reports the identification and characterization of NAT candidate in P. falciparum Pf3D7_1437000. The authors show that this protein is mainly localized in the ER and not required for protein export. However, downregulation of Pf3D7_1437000 prevents parasite development within the host red blood cell and affects the parasite's final DNA replication cycle. Most of these results are in line with the recent characterization of the homologous protein in T. gondii (TgNAT8) (Nyonda et al. 2022).

Overall, this is a nice and clear manuscript that contributes to an ER resident NAT's assessment of several biological functions of the parasite.

Most of the presented experiments appear accurately performed and well-presented, but the manuscript shows some limitations.

Major concerns

1) The most significant limitation of the study is the lack of N-terminomics data. We understand that specific pipelines are needed (available however) for the enrichment of N-termini and quantification of the N-terminal acetylation, but the conclusion cannot come

© 2023. Published by The Company of Biologists under the terms of the Creative Commons Attribution License (https://creativecommons.org/licenses/by/4.0/).

from the presented mass spectrometry data. I do not suggest to purify ER prior of MS, but a global Nterminomics analysis must be presented for WT and KD samples.

2) The presented MS data is the weakest part of the manuscript and their presentation needs to be improved to meet the mass spectrometry standards. For instance, all MS spectra need to be deposited in a public repository (eg., PRIDE) and give the access number. Furthermore, it is necessary to add a complete excel table with the recovered peptides (including the few N-terms), position, localization, protein accession, N-terminal acetylation yield.. Figure 6D is useless except to point out the very low number of retrieved N-termini which cannot allow any conclusions to be drawn. This limitation need to be better explained in the result section and avoid any overstatement (e.g., this suggests that Pf3D7_1437000 is not the NAT that acetylates PEXEL proteins ect). All the hypotheses proposed in the discussion appear valid. However, without more experiments, the argument that Pf3D7_1437000 is not relevant for ER acetylation is purely speculative and unproven.

Minor comments

1) The introduction needs to be updated and it is regrettable that the authors do not cite and introduce already in this section recent advances in the field, making a fast conclusion that the identity of PEXEL remains to be determined.
2) I got lost where Supp. Fig. 2 is reporte in the text.
3) As for the NAT localization experiments, I'm a little confused about the claimed results. I can see the location of the ER, despite the unclear merging with the PMV stain. However, I see some of them merge with the localization of aldolase (cytosol). Please clarify the author's conclusion of this part. I would also suggest mentioning the localization of TgNAT8 from the recent Nyonda et al. 2022, in line with an ER localization.
4) Among the different explanations in the discussion, the authors do not consider the possibility that N-terminal acetylation of secretory proteins may occur after ER transit and cleavage of N-terminal PEXEL along the entire secretory pathway.

Reviewer 3

Advance summary and potential significance to field

In this manuscript the authors test the hypothesis that a predicted ER resident N-acetyltransferase is responsible for acetylation of the nascent N-termini of parasite exported proteins. The authors first perform a bioinformatics approach to identify likely ER_resident NATs and choose one candidate that contains 2 putative transmemberane domains, which could guide the NAC into the secretory pathway. Using transgenic parasite lines they first confirm an ER-localisation and show an essential function for their candidate protein. However, using protein pull down and mass-spectrometry they are unable to assert an effect of NAC deletion/ depletion on the N-acetylation of 2 exported proteins, argueing that their candidate is not the ER NAT responsible for exported protein acetylation.

This study is carefully performed and well controlled and provides a solid, but negative, result for the hypothesis that their candidate is the NAT for exported proteins. While this study is important and allows the field to focus on other putative NACs, I feel it lacks a conceptual advance in our understanding how N-acetylation occurs in the ER, and what NAT is potentially responsible.

So while I am conviced that that this protein is not the exported protein NAT the field has long been looking for, the significance to the field is more limited.

Comments for the author

While the authors are generally cautions about strong statements, I do feel that there are several points which would have to be experimentally substantiated, or the authors should revise some statements.

- The authos have not shown that this protein is able to acetylate proteins. Therefore the title is slightly misleading in the absence of an assay that shows NAC activity of the protein.

© 2023. Published by The Company of Biologists under the terms of the Creative Commons Attribution License (https://creativecommons.org/licenses/by/4.0/).

- The authors have not tested the topology of the protein and whether the putative NAC is within, or outside the ER lumen. Proteinase K protection assays would clarify this.

First revision

Author response to reviewers' comments

Reviewer Comments for Polino, Hasan, et al.

We thank the reviewers for their time and effort in reviewing our manuscript. In response, we have added material to Figure 1 as well as new Supplementary Figures 1 and 6, and a Supplementary File detailing our mass spectrometry results. Below are summarized the reviewers' stated concerns alongside our responses (in purple). We hope you find our revised manuscript satisfactory.

Reviewer 1:
This paper is interesting, addresses a long-standing question in the malaria field, is very well written, and provides a balanced overview over the literature relevant to this investigation. The data presented is of high quality and largely supports the authors' conclusions.

Reviewer 1 Comments for the Author:
Nevertheless I have two major suggestions:

1. The charge distribution flanking the transmembrane domains determined the orientation of proteins targeted to the ER by transmembrane helices. There are a number of freely available prediction programmes that use this parameter to predict the topology of membrane spanning proteins in the ER. The authors should include a topology prediction of the Pf3D7_1437000 protein to see whether the NAT domain is even located in the lumen of the ER.

Thank you for directing our attention to these programs. We have now used TOPCONS to predict the membrane topology of Pf3D7_1437000. It predicts that the bulk of the protein, including the GNAT domain, faces the ER lumen. We feel that it strengthens our argument on following up on Pf3D7_1437000 as the NAT for PEXEL proteins. We have included the result in figure 1C and a new Supp. Fig 1 and discussed it in the result section on line 73 to 75.

2. With the respect to the 'bleb' localization of the Pf3D7_1437000 protein this might indicate that a major fraction of the protein is misfolded in the parasites. What do the authors know about expression levels of the untagged protein? Is their construct significantly overexpressed? Or is it possible that any of the modifications introduced affect folding? If the majority of the expressed protein were misfolded and therefore non-functional, this might explain why the relatively moderate depletion down to 40% is already lethal. The authors need to address this issue.

We thank the reviewer for this insightful comment. We do not have a quick way to compare the folding of the native and the tagged protein or to compare their levels in the cells. As we are modifying the native locus, we do not anticipate changes in the transcription level. Our conclusion from the data was that the parasite is highly sensitive to Pf3D7_1437000 depletion, which is why the 60% reduction is lethal. We find the reviewer's alternative explanation to be also plausible and therefore have included this in the discussion section at line 220-226.

Minor comments:

-page numbers are missing

Added
-n numbers in Fig. 4B are too small to read
Increased text size

© 2023. Published by The Company of Biologists under the terms of the Creative Commons Attribution License (https://creativecommons.org/licenses/by/4.0/).

-both in the abstract and on the first page of the introduction the authors say PEXEL proteins are 'translated into the ER'. This has not formally been proven. In fact, proteins in which the PEXEL signal was reduced by site-directed mutagesesis to the plasmepsin V-cleaved form and preceded by a conventional ER targeting signal sequence led to the proteins accumulating in the parasitophorous vacuole, not in the red blood cell; so while PEXEL proteins are targeted to and translated at the ER membrane, whether they are actually translocated into the ER lumen remains unclear. It would be better to rephrase these sentences.

Our understanding is that the PEXEL proteins are first loaded into the parasite ER, then travel to the parasite plasma membrane, and are released into the parasitophorous vacuole (PV). From the PV, they are exported out into the RBC cytoplasm through the PTEX complex. PV-resident proteins follow the same route, except that they are not exported out to the RBC. There have been no reports of PEXEL proteins bypassing the PV to reach the RBC, and knockdown of PTEX component EXP2 and HSP101 result in the PEXEL proteins accumulating in the PV. If the PEXEL proteins are not loaded in the ER lumen, we cannot envision a route for them to reach PV and to our knowledge, no experimental evidence exists for any alternative route. Nevertheless, the reviewer's point is well taken and we have qualified this statement in the abstract and introduction.

As for the experiment mentioned in this comment (proteins in which the PEXEL signal was reduced by site-directed mutagesesis to the plasmepsin V-cleaved form and preceded by a conventional ER targeting signal sequence led to the proteins accumulating in the parasitophorous vacuole), we are actively working to determine the reason behind this observation in a separate project. But the very fact that those constructs also end up in the PV suggests that they are also first loaded into the parasite ER lumen.

Reviewer #2 Comments:
The manuscript by Polino et al. reports the identification and characterization of NAT candidate in P. falciparum Pf3D7_1437000. The authors show that this protein is mainly localized in the ER and not required for protein export. However, downregulation of Pf3D7_1437000 prevents parasite development within the host red blood cell and affects the parasite's final DNA replication cycle. Most of these results are in line with the recent characterization of the homologous protein in T. gondii (TgNAT8) (Nyonda et al. 2022).Overall, this is a nice and clear manuscript that contributes to an ER resident NAT's assessment of several biological functions of the parasite. Most of the presented experiments appear accurately performed and well-presented, but the manuscript shows some limitations.

Major concerns
1) The most significant limitation of the study is the lack of N-terminomics data. We understand that specific pipelines are needed (available however) for the enrichment of N-termini and quantification of the N-terminal acetylation, but the conclusion cannot come from the presented mass spectrometry data. I do not suggest to purify ER prior of MS, but a global N-terminomics analysis must be presented for WT and KD samples.

Thank you for suggesting this experiment. We also considered performing N-terminomics but decided against it for this manuscript for the following reasons:

First, as mentioned in this comment, performing the experiment requires developing specific pipelines for data generation and analysis. It would require substantive investment of time and money to perform that analysis. However, we started this project to identify the NAT that acetylates mature PEXEL proteins and the potential role of that acetylation on protein export. The *P. berghei* N- terminomics dataset (Nyonda, et al. 2022) could only identify 3 processed PEXEL N-termini, highlighting the challenges and limitations of this experiment for our purpose. Therefore, before moving ahead to comprehensively characterize the N-terminal acetylation activity of Pf3D7_1437000, we decided to check whether protein export to RBC is affected and if the N-terminal acetylation status of specific PEXEL proteins is altered. Had we obtained positive data for either of those two experiments, we would have moved ahead to do N-terminomics of the WT and KD to determine the proteins that are differentially acetylated. However, as shown in the manuscript, Pf3D7_1437000 KD did not lead to protein export

© 2023. Published by The Company of Biologists under the terms of the Creative Commons Attribution License (https://creativecommons.org/licenses/by/4.0/).

defect, and the two PEXEL reporters that we could test did not have any alteration in their N terminal acetylation status.

Our conclusion from these results is that Pf3D7_1437000 is likely not the N-acetyl transferase that acetylates all PEXEL proteins. It is possible that Pf3D7_1437000 acetylates other proteins in the ER and that a global N-terminomics experiment might identify them. However, we feel the conclusion we draw from the presented set of experiments can stand on its own without the additional data. We have been conscious to not overstate our conclusion, which is limited in terms of identifying potential substrates of Pf3D7_1437000, but important in terms of identifying the NAT that acetylates PEXEL proteins, which is a known phenomenon for quite a long time now without knowledge of the identity of the gene that does it.

We have made some modifications to the manuscript to further clarify our claims and shortcomings in line of this comment. Please check line 22-24, and 264-269. We hope that these modifications will be satisfactory responses to this thoughtful concern.

2) The presented MS data is the weakest part of the manuscript and their presentation needs to be improved to meet the mass spectrometry standards. For instance, all MS spectra need to be deposited in a public repository (eg., PRIDE) and give the access number. Furthermore, it is necessary to add a complete excel table with the recovered peptides (including the few N-terms), position, localization, protein accession, N-terminal acetylation yield.. Figure 6D is useless except to point out the very low number of retrieved N-termini which cannot allow any conclusions to be drawn. This limitation need to be better explained in the result section and avoid any overstatement (e.g., this suggests that Pf3D7_1437000 is not the NAT that acetylates PEXEL proteins etc). All the hypotheses proposed in the discussion appear valid. However, without more experiments, the argument that Pf3D7_1437000 is not relevant for ER acetylation is purely speculative and unproven.

Thank you for pointing out the need for better presentation of the Mass spec data. We have made the following changes in our data presentation in response to this comment:

- **We have submitted the bottom-up mass spectrometry data (previously Fig 6D, Now Supp. Fig 6) in the Pride database and have provided the accession number (line 485)**
- **A complete Excel table has been added as Supplementary File 1.**
- **The data from the first replicate from the previous figure 6D has been withdrawn due to the low number of detected peptides.**
- **In figure 6D, we replaced the bottom-up proteomics identification of acetylated HRP2 and HRP3 with data from a top-down proteomics identification of the same proteins. A gel image for that experiment and complete mass spectrum is also presented as Supp. Fig 5.**
- **We have put the bottom-up proteomics data in a new Supp. Fig 6 where we have shown the coverage of the target proteins along with a table showing that only N-terminally acetylated peptides were detected in that experiment.**

We respectfully disagree that our conclusion is purely speculative. We have performed multiple mass spectrometry experiments on two reporter PEXEL proteins, and have not identified unacetylated forms in any of the samples. For the bottom-up proteomics approach (Supp Fig. 6), we did not enrich the N-terminal peptide using any reagent before mass spectrometry. Based on in-silico fragmentation pattern, we used Glu-C for in-gel digestion of HRP2 and HRP3 to improve the likelihood of detecting N- terminal fragments. We have detected four N-terminal HRP2 fragments from the WT and three from the Pf3D7_1437000-depleted parasites, as well as four and three N-terminal fragments for HRP3 from the same lines. All those fragments were N-terminally acetylated, even when Pf3D7_1437000 was knocked down. From our experience, the low number of detected N-terminal fragments in these experiments was as expected (see also Nyonda, et al. 2022, results referenced on line 184). We have been careful throughout to avoid overstatement in interpreting our results. Yet we believe our data suggest Pf3D7_1437000 is unlikely to acetylate HRP2 and HRP3, and that this data should be shared with the field.

Minor comments

1) The introduction needs to be updated and it is regrettable that the authors do not cite and introduce already in this section recent advances in the field, making a fast conclusion that the

© 2023. Published by The Company of Biologists under the terms of the Creative Commons Attribution License (https://creativecommons.org/licenses/by/4.0/).

identity of PEXEL remains to be determined.
We have now referenced Nyonda et al., 2022 in the introduction section at line 47-49. We thank the reviewer for this suggestion.

2) I got lost where Supp. Fig. 2 is reported in the text.
Sorry for this omission. Now added in line 84 and 88. Note that it is now Supp Fig 3 due to addition of a new Supp Fig. 1.

3) As for the NAT localization experiments, I'm a little confused about the claimed results. I can see the location of the ER, despite the unclear merging with the PMV stain. However, I see some of them merge with the localization of aldolase (cytosol). Please clarify the author's conclusion of this part. I would also suggest mentioning the localization of TgNAT8 from the recent Nyonda et al. 2022, in line with an ER localization.
The partial merging of the NAT signal with aldolase is a result of using epifluorescence microscopy, where signals from out of focus planes seem to occupy the same location. As the level of colocalization was quite clear with the ER marker PM V, we conclude that the protein is likely ER- resident. We have also mentioned the localization of TgNat8 from Nyonda et al. 2022 at line 72-73.

4) Among the different explanations in the discussion, the authors do not consider the possibility that N- terminal acetylation of secretory proteins may occur after ER transit and cleavage of N-terminal PEXEL along the entire secretory pathway.
Previously published work has shown that in *P. falciparum,* N-terminal acetylation occurs even if exported proteins were sequestered in the ER by brefeldin A treatment (blocking anterograde traffic from the ER) or addition of an ER retention signal (Chang et al., 2008; Osborne et al., 2010), suggesting it occurs prior to exit from the ER. We have mentioned that at line 41-43.

Reviewer 3:
Advance Summary and Potential Significance to Field:

This study is carefully performed and well controlled and provides a solid, but negative, result for the hypothesis that their candidate is the NAT for exported proteins. While this study is important and allows the field to focus on other putative NACs, I feel it lacks a conceptual advance in our understanding how N-acetylation occurs in the ER, and what NAT is potentially responsible. So while I am convinced that that this protein is not the exported protein NAT the field has long been looking for, the significance to the field is more limited.

Reviewer 3 Comments for the Author:

While the authors are generally cautions about strong statements, I do feel that there are several points which would have to be experimentally substantiated, or the authors should revise some statements.

- The authors have not shown that this protein is able to acetylate proteins. Therefore the title is slightly misleading in the absence of an assay that shows NAC activity of the protein.
Thank you for pointing this out. We have changed the title accordingly.

- The authors have not tested the topology of the protein and whether the putative NAC is within, or outside the ER lumen. Proteinase K protection assays would clarify this.
We have tried and failed to perform Proteinase K protection assays that separate ER from cytosolic fractions in *P. falciparum*. We have now added an in-silico prediction of the topology (Fig. 1C and Supp Fig. 1) to address this issue.

© 2023. Published by The Company of Biologists under the terms of the Creative Commons Attribution License (https://creativecommons.org/licenses/by/4.0/).

Second decision letter

MS ID#: JOCES/2022/260551

MS TITLE: An essential ER-resident N-acetyltransferase ortholog in Plasmodium falciparum

AUTHORS: Alexander J Polino, Muhammad M Hasan, Katherine Floyd, Yolotzin Avila-Cruz, Yujuan Yang, and Daniel E. Goldberg
ARTICLE TYPE: Research Article

We have now reached a decision on the above manuscript.

To see the reviewers' reports and a copy of this decision letter, please go to: https://submit-jcs.biologists.organd click on the 'Manuscripts with Decisions' queue in the Author Area. (Corresponding author only has access to reviews.)

As you will see, the reviewers gave somewhat mixed reports and I have been debating how to proceed. After much thought, I would like to see your response to the reviewers comments (for example you might want to change the text in response to review 1's point)because after which I would like to be able to accept your paper.

Please ensure that you clearly highlight all changes made in the revised manuscript. Please avoid using 'Tracked changes' in Word files as these are lost in PDF conversion.

I should be grateful if you would also provide a point-by-point response detailing how you have dealt with the points raised by the reviewers in the 'Response to Reviewers' box. Please attend to all of the reviewers' comments. If you do not agree with any of their criticisms or suggestions please explain clearly why this is so.

Reviewer 1

Advance summary and potential significance to field

The authors have addressed most of my previous queries satisfactorily.

Comments for the author

There are certainly known routes for proteins to the eukaryotic cell surface that bypass translocation into the ER and I would strongly suggest that the authors familiarize themselves with the literature rather than assuming that any protein outside that cell must have passed through the ER lumen.

The revised manuscript is now suitable for publication by JCS.

Reviewer 2

Advance summary and potential significance to field

-

Comments for the author

In this report, Polino et al. investigate a putative ER-resident N-terminal acetyl transferase (NAT), Pf3D7_1437000, in Plasmodium falciparum. The authors provided evidence that this protein is localized in the ER, is required for parasite growth, and its depletion prevents parasite development within host red blood cells and prevents parasites from completing genome replication. However, this protein has been shown to be unimportant for protein export and acetylation of two exported proteins HRP2 and HRP3. This is an interesting short report which in

© 2023. Published by The Company of Biologists under the terms of the Creative Commons Attribution License (https://creativecommons.org/licenses/by/4.0/).

my opinion can give a broader perspective on studies of GNAT proteins in parasites. However, this work failed to answer the initial question regarding the possibility that Pf3D7_1437000 could be a NAT and in particular the NAT involved in N-terminal acetylation (NTA) of secreted proteins.

Main points:

The main claim of the present manuscript is that Pf3D7_1437000 is not the enzyme responsible for the NTA of secreted proteins in Plasmodium falciparum. However, in support of this claim, the authors tested the NTA status of only two secreted proteins. To conclude with this statement, the authors need to perform a series of additional control experiments:
1) The authors should test in vitro the ability of Pf3D7_1437000 to perform an NTA activity or eventually a KAT activity.
2) The authors need to perform a more extensive N-terminomics analysis instead of focusing on just two proteins.
3) The experiment related to Figure 6D should be performed with at least three biological replicates. 3) The title should be changed as it does not reflect the results presented (no evidence on NTA activity of Pf3D7_1437000 is provided).

Minor points:
1) Mass specification data must be deposited in a public MS repository and available to reviewers with code and password.
2) The N-terminus of HRP2 and HRP3 should be reported in a figure and discussed whether or not they could be recovered in an MS experiment.
3) Figure 6D should be more precise and include a more detailed corresponding legend (e.g., information about the recovered acetylated peptides, as well as the IDs and locations of the corresponding proteins). Also, the text in the results section is better discussed, accordingly.

Reviewer 3

Advance summary and potential significance to field

Overall the study is carefully performed and the authors have reflected one concern and changed the title regarding the predicted NAT activity, and topology.

Comments for the author

I still be believe that the study is important for the exclusion of this putative NAT for PEXEL protein acetylation, but that the conceptual advance for the field is limited.

Second revision

Author response to reviewers' comments

Reviewer 1 Advance Summary and Potential Significance to Field:
The authors have addressed most of my previous queries satisfactorily.

Reviewer 1 Comments for the Author:
There are certainly known routes for proteins to the eukaryotic cell surface that bypass translocation into the
ER and I would strongly suggest that the authors familiarize themselves with the literature rather than assuming that any protein outside that cell must have passed through the ER lumen.

The revised manuscript is now suitable for publication by JCS.

Response: Thank you for the suggestion. We have now included a potential alternative explanation (line 209-213) in the discussion section. We have also mentioned potential alternative routes for parasite proteins to RBC cytoplasm (line 254-256).

© 2023. Published by The Company of Biologists under the terms of the Creative Commons Attribution License (https://creativecommons.org/licenses/by/4.0/).

Reviewer 2 Comments for the Author:

Comments to the Author

In this report, Polino et al. investigate a putative ER-resident N-terminal acetyl transferase (NAT), Pf3D7_1437000, in Plasmodium falciparum. The authors provided evidence that this protein is localized in the ER, is required for parasite growth, and its depletion prevents parasite development within host red blood cells and prevents parasites from completing genome replication. However, this protein has been shown to be unimportant for protein export and acetylation of two exported proteins HRP2 and HRP3. This is an interesting short report which in my opinion can give a broader perspective on studies of GNAT proteins in parasites.
However, this work failed to answer the initial question regarding the possibility that Pf3D7_1437000 could be a NAT and in particular the NAT involved in N-terminal acetylation (NTA) of secreted proteins.

Main points:

The main claim of the present manuscript is that Pf3D7_1437000 is not the enzyme responsible for the NTA of secreted proteins in Plasmodium falciparum. However, in support of this claim, the authors tested the NTA status of only two secreted proteins. To conclude with this statement, the authors need to perform a series of additional control experiments:

1) The authors should test in vitro the ability of Pf3D7_1437000 to perform an NTA activity or eventually a KAT activity.

This is a worthwhile project but is outside the scope of our thesis that Pf3D7_1437000 is not the PEXEL NAT. Nonetheless, the current title of our article reflects our ambiguity about Pf3D7_1437000's catalytic function.

2) The authors need to perform a more extensive N-terminomics analysis instead of focusing on just two proteins.

This will be essential for defining the potential substrates of Pf3D7_1437000. However, that is not goal of this manuscript. We have discussed the utility of N-terminomics for any potential follow-up studies in line 265-271.

3) The experiment related to Figure 6D should be performed with at least three biological replicates.

We have already performed the mass spectrometry experiments 6 times, twice each with the knockdown and knockout parasites using undigested protein samples and twice with Glu-C digested protein samples.

4) The title should be changed as it does not reflect the results presented (no evidence on NTA activity of Pf3D7_1437000 is provided).

We have already changed the title to reflect that we do not have definitive data on Pf3D7_1437000's catalytic activity.

Minor points:

1) Mass specification data must be deposited in a public MS repository and available to reviewers with code and password.

The Glu-C digested mass spectrometry data were submitted to the PRIDE database, with accession number PXD038231. Please use reviewer_pxd038231@ebi.ac.uk and hTmEePE2 for username and password.

2) The N-terminus of HRP2 and HRP3 should be reported in a figure and discussed whether or not they could be recovered in an MS experiment.

© 2023. Published by The Company of Biologists under the terms of the Creative Commons Attribution License (https://creativecommons.org/licenses/by/4.0/).

They are reported in Fig S6

3) Figure 6D should be more precise and include a more detailed corresponding legend (e.g., information about the recovered acetylated peptides, as well as the IDs and locations of the corresponding proteins). Also, the text in the results section is better discussed, accordingly.

Figure 6D is the whole protein proteomics spectra and we only identified the acetylated protein.

Reviewer 3 Advance Summary and Potential Significance to Field:

Overall the study is carefully performed and the authors have reflected one concern and changed the title regarding the predicted NAT activity, and topology.

Reviewer 3 Comments for the Author:

I still be believe that the study is important for the exclusion of this putative NAT for PEXEL protein acetylation, but that the conceptual advance for the field is limited.

Third decision letter

MS ID#: JOCES/2022/260551

MS TITLE: An essential ER-resident N-acetyltransferase ortholog in *Plasmodium falciparum*

AUTHORS: Alexander J Polino, Muhammad M Hasan, Katherine Floyd, Yolotzin Avila-Cruz, Yujuan Yang, and Daniel E. Goldberg
ARTICLE TYPE: Research Article

I am happy to tell you that your manuscript has been accepted for publication in Journal of Cell Science, pending standard ethics checks.

© 2023. Published by The Company of Biologists under the terms of the Creative Commons Attribution License (https://creativecommons.org/licenses/by/4.0/).
